# Breeding system diversification and evolution in American *Poa* supersect. *Homalopoa* (Poaceae: Poeae: Poinae)

**DOI:** 10.1093/aob/mcw108

**Published:** 2016-07-03

**Authors:** Liliana M. Giussani, Lynn J. Gillespie, M. Amalia Scataglini, María A. Negritto, Ana M. Anton, Robert J. Soreng

**Affiliations:** ^1^Instituto de Botánica Darwinion, San Isidro, Buenos Aires, Argentina; ^2^Research and Collections Division, Canadian Museum of Nature, Ottawa, Ontario, Canada; ^3^Universidad de Magdalena, Santa Marta, Colombia; ^4^Instituto Multidisciplinario de Biología Vegetal (IMBIV), CONICET-UNC, Córdoba, Argentina; ^5^Department of Botany, Smithsonian Institution, Washington, DC, USA

**Keywords:** Breeding system, diclinism, dioecism, DNA, gynomonoecism, gynodioecism, molecular dating, molecular phylogeny, *Poa*, *Homalopoa*, South America, North America

## Abstract

**Background and Aims** *Poa* subgenus *Poa* supersect. *Homalopoa* has diversified extensively in the Americas. Over half of the species in the supersection are diclinous; most of these are from the New World, while a few are from South-East Asia. Diclinism in *Homalopoa* can be divided into three main types: gynomonoecism, gynodioecism and dioecism. Here the sampling of species of New World *Homalopoa* is expanded to date its origin and diversification in North and South America and examine the evolution and origin of the breeding system diversity.

**Methods** A total of 124 specimens were included in the matrix, of which 89 are species of *Poa* supersect. *Homalopoa* sections *Acutifoliae*, *Anthochloa*, *Brizoides*, *Dasypoa*, *Dioicopoa*, *Dissanthelium*, *Homalopoa sensu lato* (*s.l.*), *Madropoa* and *Tovarochloa*, and the informal Punapoa group. Bayesian and parsimony analyses were conducted on the data sets based on four markers: the nuclear ribosomal internal tanscribed spacer (ITS) and external transcribed spacer (ETS), and plastid *trn*T-L and *trn*L-F. Dating analyses were performed on a reduced *Poa* matrix and enlarged Poaceae outgroup to utilize fossils as calibration points. A relaxed Bayesian molecular clock method was used.

**Key Results** Hermaphroditism appears to be pleisiomorphic in the monophyletic *Poa* supersect. *Homalopoa*, which is suggested to have originated in Eurasia 8·4–4·2 million years ago (Mya). The ancestor of *Poa* supersect. *Homalopoa* radiated throughout the New World in the Late Miocene–Early Pliocene, with major lineages originating during the Pliocene to Pleistocene (5–2 Mya). Breeding systems are linked to geographic areas, showing an evolutionary pattern associated with different habitats. At least three major pathways from hermaphroditism to diclinism are inferred in New World *Homalopoa*: two leading to dioecism, one via gynodioecism in South America and another directly from hermaphroditism in North America, a result that needs to be checked with a broader sampling of diclinous species in North America. A third pathway leads from hermaphroditism to gynomonoecism in Andean species of South America, with strictly pistillate species evolving in the highest altitudes.

**Conclusions** Divergence dating provides a temporal context to the evolution of breeding systems in New World *Poa* supersect*. Homalopoa*. The results are consistent with the infrageneric classification in part; monophyletic sections are confirmed, it is proposed to reclassify species of sect. *Acutifoliae*, *Dasypoa* and *Homalopoa s.l.* and it is acknowledged that revision of the infrageneric taxonomy of the gynomonoecious species is needed.

## INTRODUCTION

*Poa* L. (Poaceae, Pooideae, Poeae) is one of the largest genera of grasses, with an estimated 530 species distributed worldwide (Soreng *et al.*, 2007, 2015*b*; Gillespie *et al.*, 2008), mostly in high altitudes and/or latitudes of both hemispheres (Hartley, 1961). *Poa* is generally a morphologically well-marked and monophyletic genus (Soreng *et al.*, 1990, 2015*a*; Gillespie and Soreng, 2005; Gillespie *et al.*, 2007, 2008). Molecular studies have also supported uniting 11 genera with *Poa*, including six whose species are now considered members of supersect. *Homalopoa*: *Anthochloa* Nees & Meyen, *Dasypoa* Pilg., *Dissanthelium* Trin. and *Tovarochloa* T.D. Macfarl. & But from South America; and *Austrofestuca* (Tzvelev) E. B. Alexeev *sensu stricto* (*s.s.*) and *Neuropoa* W. D. Clayton from Australia (Gillespie and Soreng, 2005; Gillespie *et al.*, 2007, 2008, 2009; Jacobs *et al.*, 2008; Soreng *et al.*, 2009; Refulio-Rodriguez *et al.*, 2012). The South American genus *Aphanelytrum* (Hack.) Hack. has also been confirmed as part of the *Homalopoa* clade but has not yet been synonymized under *Poa* (Gillespie *et al.*, 2008; Refulio-Rodriguez *et al.*, 2012; P. M. Peterson and R. J. Soreng, unpubl. res.).

Recent molecular phylogenetic studies have substantially influenced the classification of *Poa*. *Poa* is currently divided into five subgenera: *Sylvestres*, *Ochlopoa*, *Pseudopoa*, *Stenopoa* and *Poa*, corresponding to the five major plastid clades in the genus (Gillespie *et al.*, 2007; updated in Gillespie *et al.*, 2008; Soreng *et al.*, 2010). The largest subgenus, *Poa*, is further divided into two clades corresponding to supersections *Poa* and *Homalopoa*. The latter was first recognized as a clade based on plastid restriction site data (Soreng, 1990; Gillespie and Soreng, 2005), and then confirmed with plastid sequence data and formally named (Gillespie *et al.*, 2007); however, when analysing nuclear ribosomal DNA (nrDNA) sequences, species of the two supersections were intermingled in a *Poa–Homalopoa* clade or resolved in an independent clade (Nosov and Rodionov, 2008; Gillespie *et al.*, 2009; Nosov *et al.*, 2015) for which no corresponding plastid type is known (‘X-clade’ of Gillespie *et al.*, 2009).

**Fig. 1.**
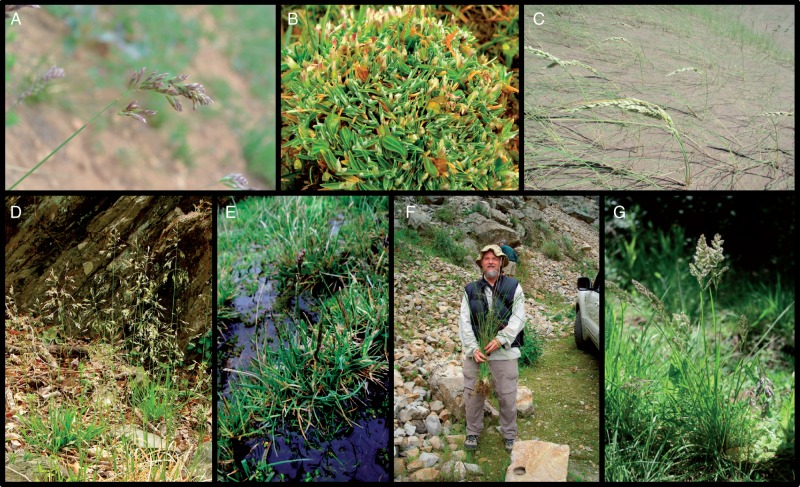
Morphological variation among species of *Poa* supersect. *Homalopoa* of the New World. (A) *Poa cusickii* subsp. *purpurascens* (clade C); (B) *P. unispiculata* (clade E); (C) *P. bergii* (clade D), photo: Daniel Testoni; (D) *P. cuspidata*; (E) *P. aequigluma* (clade E); (F) Robert Soreng holding a specimen of *P. horridula* (clade F); (G) *P. lanigera* (clade D); clades are given following the results as presented in Supplementary Data Fig. S1.

*Poa* supersect. *Homalopoa* (hereafter excluding species with X-clade nrDNA) is a large and diversified clade in terms of both species and sections, and includes about half of the species in *Poa*; it is currently divided into ten sections worldwide along with the Punapoa informal assemblage (Fig. 1; Table 1)*.* The New World represents a major centre of diversity with 144 endemic *Homalopoa* species. With many species and morphological diversity and adaptations to many niches, *P.* supersect. *Homalopoa* is difficult to characterize morphologically. Species are ephemeral annuals to long-lived perennials, ranging in size from a few centimetres to 1·5 m tall (Gillespie and Soreng, 2005), upper culm sheaths are generally fused at the base for more than a quarter of the length, the lower sheaths are commonly distinctly to strongly compressed, and lemmas are generally strongly five-veined. They occupy a wide diversity of habitats, including moist temperate deciduous forests, coniferous forests, dry steppe, arid sub-tropical deserts, pampas, paramo, puna, wet saline meadows, dunes, low-arctic meadows and acidic to basic and sometimes ultramafic substrates, among others.

**Table 1.** Classification of *Poa* subg. *Poa* supersect. *Homalopoa* (Giussani 2000; Negritto and Anton, 2000; Gillespie *et al.*, 2007; updated in Soreng *et al*., 2009; Refulio-Rodriguez *et al*., 2012)

Sections	New World/BS		Old World/BS		Worldwide	Geographical distribution
*Acutifoliae*	2	h, gd	0		2	S Andes
*Anthochloa*	1	gm	0		1	Andes
*Brizoides*	0		48	h	48	Australasia
*Dasypoa*	3	h, gm	0		3	Andes (1 sp. also in Mexico and Guatemala)
*Dioicopoa*	31	di (gd)	0		31	S South America (1 sp. S USA)
*Dissanthelium s.s.*	7	gm	0		7	Andes (1 subsp. in Mexico)
*Homalopoa s.l.* (incl. *Plicatae*)	59	h, gm, sgm (gd, di)	28	h, gm, sgm (gd, di)	87	America, Eurasia
*Madropoa*	22	di (p, gd, sgm)	0		22	North America (1 sp. in Chile *P. pfisteri*)
*Monandropoa*	1	h	0		1	S South America
Punapoa informal group	12	p, gd, gm	0		12	Andes (2 spp. in Mexico)
*Tovarochloa*	1	h	0		1	Peru
Unplaced	3	h (gm)			3	America
Genus *Aphanelytrum* (not transferred yet)	2	h	0		2	N Andes
Total	144		76		220	

Numbers of species in the Americas (New World), the Old World (including Australasia) and worldwide are given for each section, the Punapoa informal species group and *Aphanelytrum* (confirmed member of the supersect. *Homalopoa* clade, but not yet synonymized under *Poa*) (taxonomic placement data derived from Tzvelev, 1983; Probatova, 2003; Soreng *et al*., 2003; Zhu *et al.*, 2006; Soreng, 2007; Giussani *et al.*, 2012; Refulio-Rodriguez *et al.*, 2012; Soreng and Peterson, 2012).

Most frequent breeding system (BS) in sections of supersect. *Homalopoa* (infrequent to rare types in parentheses): h, hermaphrodite; gm, simple gynomonoecy; sgm, sequentially adjusted gynomonoecy; gd, gynodioecy; p, strictly pistillate; di, dioecy.

Section *Homalopoa sensu latu* (*s.l.*) is the largest, most widespread and most heterogeneous section in supersect. *Homalopoa*, and is found in both the Americas and Eurasia (Soreng, 2007). In the strict sense, sect. *Homalopoa* comprises approximately five Eurasian species [and possibly the North American diploid *P. occidentalis* (Vasey) Vasey], but remains poorly defined and may also be heterogeneous. In the Old World, only one section is endemic, sect. *Brizoides* of Australasia. Following Giussani (2000, for *Dioicopoa*), Negritto and Anton (2000, for gynomonoecious *Poa*, and sect. *Monandropoa* Parodi), Soreng *et al.* (2003), Gillespie *et al.* (2007, for sect. *Anthochloa*), Zuloaga *et al.* (2008) and Refulio-Rodriguez *et al.* (2012, for sect. *Dissanthelium* and *Tovarochloa*), eight sections are restricted to the Americas (Table 1): *Anthochloa* and *Tovarochloa* are endemic to the southern Andes; *Dasypoa* and *Dissanthelium* are primarily Andean, each with one disjunct species between the Andes and Mesoamerica [*P. scaberula* Hook. f. and *P. calycina* (J. Presl) Kunth, respectively]; *Dioicopoa* is primarily southern South American, with one species in the southern USA (*P. arachnifera* Torr.); *Monandropoa* includes a single species close to *P. scaberula*, endemic to Catamarca and Tucumán provinces of Argentina, above 3000 m; *Acutifolae* is endemic to the central Chilean–Argentinean Andes; and *Madropoa* is North American (with one Chilean species, *P. pfisteri* Soreng, tentatively placed here by Soreng and Peterson, 2008). In addition, Punapoa is recognized as an informal species group of the Andes, with two species disjunct between the Andes and Mexico (*P. chamaeclinos* Pilg. and *P*. *gymnantha* Pilg.) (Soreng *et al.*, 2003; Gillespie *et al.*, 2007; Giussani *et al.*, 2012; Soreng and Peterson, 2012). *Aphanelytrum* (Hack.) Hack., when synonymized under *Poa* (P. M. Peterson and R. J. Soreng, unpubl. res.), may represent a new section from the northern Andes.

Species of *Poa* show an exceptional diversity in breeding systems represented by the occurrence of hermaphroditism to dioecism (Parodi, 1936; Marsh, 1952; Nicora, 1978; Connor, 1979; Anton and Connor, 1995; Giussani, 2000; Negritto and Anton, 2000; Soreng, 1991; Soreng and Keil, 2003). Although hermaphroditism is the most common reproductive system among *Poa* species, dicliny was estimated to occur in about 30 % of the species (R. J. Soreng, unpubl data; based on approx. 166 diclinous species out of a total of approx. 530 *Poa* species). Most of the species diversity and variation in dicliny in *Poa* is found in sections and informal groups of *Poa* subg. *Poa* supersect. *Homalopoa* (Table 1).

While some species spread vegetatively by rhizomes forming patches or spreading, apomixis is also a common mode of asexual propagation in high polyploid species of *Poa*, partially or fully supplanting sexual reproduction to ensure the production of seed (Kelley *et al.*, 2009) or bulbils. While well known in *Poa* of the northern hemisphere (Kellogg, 1989; Kelley *et al.*, 2009), apomictic production of seed was assumed or strongly suspected in pistillate populations of Andean species of *Poa* (Anton and Connor, 1995), and first confirmed anatomically in *P. gymnantha* in South America (Negritto *et al.*, 2008). Pseudovivipary is another method of vegetative or apomictic reproduction, producing vegetative propagules (bulbils) in the spikelets instead of normal florets; in the Americas, it is mostly associated with species from higher latitude regions of extreme cold weather or areas of high rainfall in the Circumboreal biome and the Patagonian floristic regions (Moore and Doggett, 1976; Pierce *et al.*, 2003), and with differences in the onset of the rainy season (Ofir and Kigel, 2014).

**Fig. 2.**
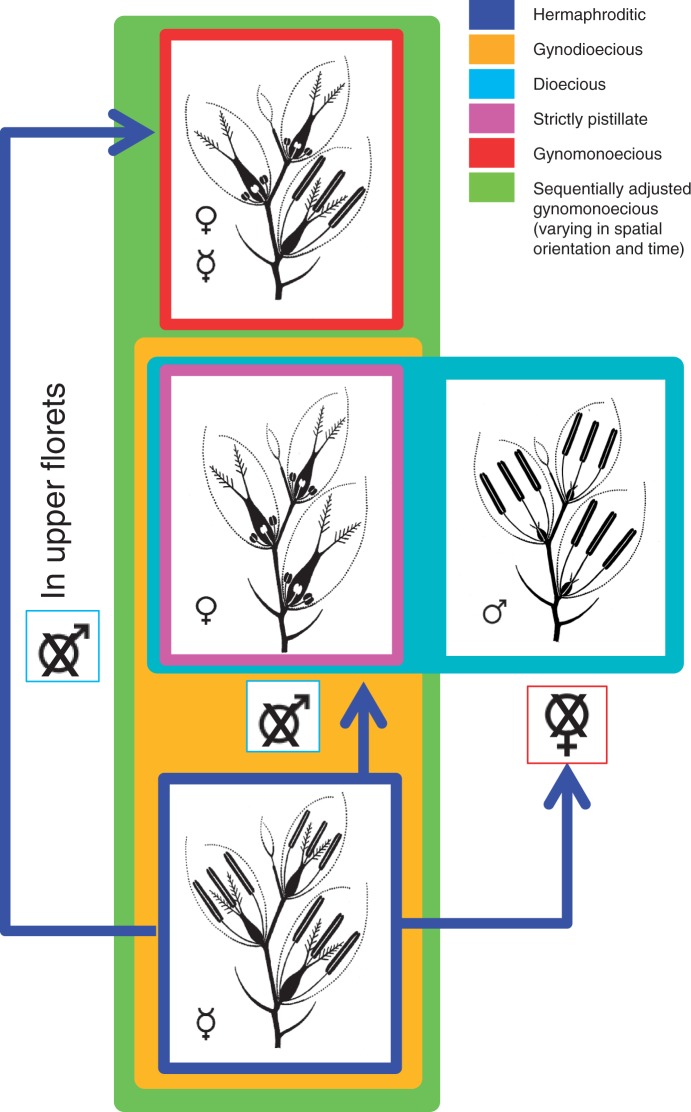
Breeding systems in *Poa* supersect. *Homalopoa*; diagram showing sexes of flowers within spikelets for each breeding system. Blue: hermaphroditic; the diagram shows flowers with well-developed pistils and stamens in all flowers of the spikelet. Yellow: gynodioecious; species having a mixture of individuals with hermaphroditic flowers and individuals with only pistillate flowers. Light blue: dioecious; represents staminate flowers and pistillate flowers separated in different individuals. Pink: represents individuals with strictly pistillate flowers in all spikelets. Red: gynomonoecious; individuals with spikelets with basal flowers hermaphroditic and upper flowers pistillate. Green: sequentially adjusted gynomonoecious; sex of flowers varying in spatial orientation and time: individuals with pistillate flowers within spikelets to whole inflorescences, or pistillate flowers produced in later inflorescences increasing through the growing season in some plants, while inflorescences remain perfect in other plants. Crosses over sex diagrams denote suppression of maleness or femaleness.

Diclinism, in the broad sense, includes any occurrence or arrangement of unisexual flowers (with or without co-occurrence of hermaphroditic flowers) within or among individuals of a species, hence including monoecy and all its sub-types (gynomonoecy, andromonoecy), androdioecy, gynodioecy, dioecy and trioecy. Figure 2 represents all types of floral arrangements found within *Poa*; dicliny is well represented in the Americas, and is also found in Asia, New Zealand and the Sub-antarctic islands (Anton and Connor, 1995; Soreng, 2007; Edgar and Connor, 2010; Giussani *et al.*, 2012). Gynomonoecy, in its simple form, where spikelets bear pistillate upper florets and perfect basal florets, is well represented among supersect. *Homalopoa* species of the informal group Punapoa, sect. *Anthochloa*, sect. *Dissanthelium*, sect*. Dasypoa* and sect. *Homalopoa s.l.* in South America (Anton, 1978; Negritto and Anton, 2000, 2006; Soreng *et al.*, 2003; Negritto *et al.*, 2008), while simple gynomonoecy is only present in non-*Homalopoa* species in North America [*P. abbreviata* R. Br. and *P. suksdorfii* (Beal) Vasey ex Piper of *P.* subg. *Stenopoa* sect. *Abbreviatae*, and non-native *P. annua* L., *P. infirma* Kunth and *P. supina* Schrad. of subg. *Ochlopoa* sect. *Micrantherae*]. As a sub-type of gynomonoecy, sequentially adjusted gynomonoecy (Soreng and Keil, 2003) is found mainly in North American sect. *Madropoa* and refers to temporal variation in the production of pistillate flowers within spikelets to whole inflorescences, the percentage of pistillate flowers produced in later inflorescences increasing through the growing season in some plants, while inflorescences remain perfect in other plants. Gynodioecious and dioecious species have sexes separated in different individuals within a population. Species having mixtures of individuals with all or mainly hermaphrodite flowers and individuals with only pistillate flowers are known as gynodioecious. Gynodioecism is relatively infrequent in *Poa*, known in only 12 species (including nine species of supersect. *Homalopoa*, all from the New World). Dioecism is the most extreme expression of sexual differentiation, with staminate flowers and pistillate flowers separated in different individuals, usually in a ratio of 1:1. In the genus *Poa*, dioecy is restricted to the Americas within supersect*. Homalopoa*, and to New Zealand (where it is confined to five species with X-clade nrDNA); sexual dimorphism developed only in species of the South American sect. *Dioicopoa*. The occurrence of dioecism implies the suppression of both maleness and femaleness in respective individuals, while all other reproductive systems in New World *Homalopoa* result from different timing and zone of action of maleness suppression (Fig. 2).

Dioecious species are present in disjunct areas in the Americas, and taxonomically grouped in two sections within supersect. *Homalopoa*: *Dioicopoa* and *Madropoa*. Morphologically, sect. *Dioicopoa* is well diversified and marked by sexual dimorphism. Florets of pistillate plants are usually abundantly pubescent, while those of staminate plants are glabrous or sparsely pubescent. Moreover, pistillate florets are generally larger and fewer per spikelet than staminate florets (Giussani, 2000; Giussani *et al.*, 2000, 2008, 2012). In addition, habitat preference favouring the establishment of pistillate individuals (Giussani *et al.*, 1996; Bertiller *et al.*, 2000, 2002) or different responses of sexes to competition with neighbours under grazing pressure (Giussani and Collantes, 1997; Graff *et al.*, 2013) have been reported. In contrast, dimorphism between sexes in the North American dioecious sect. *Madropoa* has not been found or is negligible (Soreng, 1991). Although dioecy has presumably originated at least three times within *Poa* (based on the tree in Gillespie *et al.*, 2009), the monophyly and origins of sect. *Dioicopoa* and sect. *Madropoa* are still in need of clarification. The relationship of dioecious species to gynodioecious and gynomonoecious species could reveal possible ancestral pathways to dioecy (Anton and Connor, 1995).

Cytogenetically, *Poa* is characterized in having medium to large sized chromosomes with a base number of *x* = 7 and a high frequency of polyploid species. Only about 9 % of the reported species are diploids, with an additional 4–6 % having both diploid and polyploid populations, mostly from Europe and few from Asia (Moore, 1982; Rodionov *et al.*, 2010; Soreng *et al.*, 2010). Only three diploid species (2*n* = 14), *P. lettermanii* Vasey, *P. pseudoabbreviata* Roshev. (sect. *Abbreviatae*) and *P. occidentalis* (sect. *Homalopoa*), are native to North America, and none has been reported from South America (apart from the introduced diploid species *P. infirma*, *P. supina* and *P. trivialis* L.). In the southern hemisphere, only tetraploid and octoploid to high polyploid species have been reported (Hair and Beuzenberg, 1961; Hunziker, 1978; Murray *et al.*, 2005); in South America, dioecious species were found to have 2*n* = 28 or 56 (Saura, 1943, 1948; Bowden and Senn, 1962; Hunziker, 1978; Guillin *et al.*, 1995), while an exclusively pistillate species, *P. gymnantha*, was counted as 2*n* = 70 (Negritto *et al.*, 2008).

Taking into account reproductive systems of species, and the distribution of high frequency of diploids, the origin of the genus was suggested to have been in Eurasia, with hermaphrodite species arriving in North America via Beringia (Soreng, 1990; Anton and Connor, 1995), and then diversifying into different gynomonoecious, gynodioecious and dioecious lineages in North and South America. The most relevant hypotheses on the evolution of sexes within *Poa* have been presented by Anton and Connor (1995), who postulated that after migrating to North America, part of the migrating hermaphrodite species of *Poa* derived to dioecism, probably via gynodioecism, and to a much lesser extent from hermaphrodite to gynomonoecism. In South America, they also postulated that part of the migrating hermaphrodite species of *Poa* differentiated into gynomonoecism, with apomictic pistillate populations evolving in some alpine species, while another part of migrating hermaphrodite species of *Poa* independently differentiated into gynodioecism, from which dioecism evolved in the same area. Few hypotheses have been offered to relate any geological or climatological events to the origin and diversification of *Poa*. In Australasia, *Poa* is suggested to have diversified within the last 4·3 million years, correlating with the appearance of grasslands in the mid-Pliocene (Birch *et al.*, 2014), and a recent rapid radiation was reported for the Australian alpine *Poa* species (Griffin and Hoffmann, 2014). However, no time for the arrival and diversification of the breeding system has been postulated for species in the Americas.

While the phylogeny of the genus *Poa* has been investigated in previous studies and the taxonomy has been generally sorted out, we here increase sampling to understand the origin and diversification of *Poa* supersect. *Homalopoa* and its breeding systems in North and South America. Dating of major phyletic nodes in *Poa* is hampered by the absence of fossils; hence, to date divergence time within supersect. *Homalopoa*, we selected stratigraphically well-dated and taxonomically identifiable micro- and macrofossils of the Poaceae family for calibration points. Molecular phylogenetic tools offer an independent source to test evolutionary hypotheses and to estimate dates on the origin of major lineages. Our aims are to analyse relationships among species of supersect. *Homalopoa* present in the Americas and the Southern Hemisphere: sections *Acutifoliae*, *Anthochloa*, *Brizoides*, *Dasypoa*, *Dioicopoa*, *Dissanthelium*, *Homalopoa*, *Madropoa* and *Tovarochloa* and the Punapoa informal group; and to date principal nodes within *Poa* supersect. *Homalopoa*. Hypotheses concerning the patterns of evolution of diclinous breeding systems in *Poa* are evaluated based on our phylogenetic results.

## MATERIALS AND METHODS

### DNA isolation, amplification and sequencing

Plants were field collected and dried in silica gel, and DNA was extracted using the modified CTAB (cetyltrimethylammonium bromide) protocol from Doyle and Doyle (1987). DNeasy Plant Mini Kits (Qiagen) were used to extract DNA from herbarium specimens when fresh material was not available. Alternatively, the silica-based column method of Alexander *et al.* (2007) was used for both silica gel dried and herbarium material.

A preliminary analysis on the variability of different markers was evaluated. A total of six markers: ITS and ETS (nuclear), and *trn*T-L, *trn*L-F, *rpo*A and *rpl*16 (plastid) were sequenced and analysed using parsimony. Only the most informative regions were then selected. Subsequently, two nuclear ribosomal markers, ETS and ITS, and two plastid regions, *trn*T-L and *trn*L-F, were amplified and sequenced for every sample. The ETS fragment (comprising the 3′ region of the external transcribed spacer of 18S–26S rDNA) was amplified using the forward primer RETS4 designed by Gillespie *et al.* (2010) and the reverse primer 18S-IGS (Baldwin and Markos, 1998) or 18S-R (Starr *et al.*, 2003). The ITS region, including the internal transcribed spacers ITS 1 and ITS 2 and the 5·8S rDNA gene, was amplified with primers designed by White *et al.* (1990) using ITS5 as the forward primer and ITS4 as the reverse primer, or using the primer combination KRC (Torrecilla and Catalán, 2002) and AB102 (Douzery *et al.*, 1999). Internal primers ITS2 and ITS3 were also used when sequences were difficult to amplify.

The *trn*T-L and *trn*L-F regions were amplified using the primer pairs denoted by a-b, c-d, and e-f as described by Taberlet *et al.* (1991). These primers amplify the spacer region between the *trn*T(UGU) and *trn*L(UAA) 5′ exon (i.e. the *trn*T-L spacer), the intron of the *trn*L(UAA) 5′ exon and the *trn*L(UAA) 3′ exon (i.e. the *trn*L intron), and the spacer region between the *trn*L(UAA) 3′ exon and the *trn*F(GAA) exon (i.e. the *trn*L-F spacer), respectively.

Polymerase chain reactions were performed in a 25 μL final volume with 50–100 ng of template DNA, 0·2 μm of each primer, 25 μm of DNTPs, 5 mm MgCl_2_, 1× buffer and 1·5 U of *Taq* polymerase (Invitrogen). The reaction conditions were: a first period of denaturation at 94 ºC for 5 min, followed by 35 cycles of denaturation at 94 ºC for 30 s, annealing at 48 ºC (52 ºC for ITS) for 1 min and extension at 72 ºC for 90 s. Final extension at 72 ºC for 6 min terminated the reactions. PCR products were run out on a 1 % TBE agarose gel, stained with SYBR safe™ DNA gel stain (Invitrogen) and visualized in a blue light transilluminator. Automated sequencing was performed by Macrogen, Inc. Alternatively, PCR and sequencing followed the methods of Gillespie *et al.* (2008, 2009). Both strands were sequenced for each fragment.

### Phylogenetic sampling

A total of 124 specimens were included in the matrix; 116 represent the genus *Poa*, of which 105 are specimens of *Poa* supersect. *Homalopoa* belonging to a total of 89 species. We here included species of sections *Acutifoliae*, *Anthochloa*, *Brizoides, Dasypoa*, *Dioicopoa*, *Dissanthelium*, *Homalopoa*, *Madropoa*, *Tovarochloa* and the Punapoa group (Supplementary Data Appendix S1). *Aphanelytrum* was represented by two species. Outgroups were selected to include representatives of all other subgenera of *Poa* known from the Americas: subg. *Poa* sects *Macropoa* and *Poa*, subg. *Ochlopoa* sects *Alpinae* and *Parodiochloa*, subg. *Stenopoa* sects *Pandemos*, *Secundae* and *Stenopoa*, and subg. *Sylvestres*. To anchor *Poa*, we added species of the following genera: *Alopecurus* (sub-tribe Alopecurinae), *Arctagrostis*, *Arctophila*, *Nicoraepoa* (sub-tribe Poinae *s.l.*) and *Phleum* (sub-tribe Phleinae) (following Soreng and Gillespie, 2007; Gillespie *et al.*, 2008, 2010)*.* For classification purposes, we followed the most current taxonomy for each taxon (Table 1; Appendix S1).

### Phylogenetic analyses

Sequence editing and assembly were performed using Chromas Pro ver 1·7·6 (Technelysium Pty Ltd, South Brisbane, Queensland, Australia) and BioEdit version 5·0·9 (Hall, 1999) or Geneious vers. 6·1·5 (Biomatters Ltd., http://www.geneious.com). The whole data set was aligned with MAFFT ver. 7 (Katoh and Standley, 2013) and the alignment was then manually checked. When amplification failed for a fragment, the respective positions were coded as missing data in separate or combined matrices. Percentages of missing data (gaps not included) were calculated for each matrix (Table 2).

**Table 2.** Information on sequences and matrices derived from the ETS and ITS ribosomal nuclear regions, and *trn*T-L-F plastid markers, and the combined (ETS + ITS + *trn*T-L-F) data set

	ETS	ITS	*trn*T-L-F	Combined
No. of taxa	124	122 without *P. pfisteri/P. schizantha*	124	124
Length of the alignment	560	685	2204	3449
*Poa* shortest sequence	527 bp = *P. apiculata*	665 bp = *P. interior*	1751 bp (=*P. holciformis* + *P. trivialis*)	–
*Poa* longest sequence	544 bp = *P. trivialis*	670 bp = *P. cucullata*	1883 bp (*P. alopecurus* subsp. *alopecurus*)	–
Missing data (% )	1·9	4·7	2·8	3
No. of informative characters	157	135	125	417
Length of the shortest trees*	430	380	223	1144
Parsimony indexes	Ci = 0·52, Ri = 0·82	Ci = 0·51, Ri = 0·74	Ci = 0·67, Ri = 0·87	Ci = 0·49, Ri = 0·76
No. of nodes in the strict consensus tree	32	30	31	61
No. of nodes with bootstrap support >50 %	36	26	33	54
Unambiguous informative indels within *Poa*	1 bp insertion = T (*P. mendocina*+ both specimens of *P. hachadoensis*); 1 bp insertion = A (*P*. *diabolii* + *P. douglasii* + *P. macrantha*); 2 bp insertion = AA (*P. glauca* + both *P. hachadoensis* + *P. interior* + *P.mendocina* + *P. pratensis* spp. *irrigate* + *P. pratensis* spp. *alpigena* + *P. secunda* + *P.stenantha* + *P.trivialis*)	1 bp insertion = G (*P. cucullata* + *P. alpina*) 1 bp insertion = T (*P. chamaechlinos* + *P. flabellata*) 1 bp = A (*P. aequigluma* + *P. aequatoriensis*) 1 bp = G (*P. aequatoriensis* + *P. chamaechlinos*) 2 bp deletion (*P. glauca* + *P. interior* + *P. secunda* + *P. stenantha*	1 bp insertion = A (*P. cucullata* + *P. pratensis*) 6 bp insertion = TAACTT (*Homalopoa* + *P. hachadoensis* var. *hachadoensis* + three subspecies of *P*. *pratensis)* 2 bp insertion = AT (*P. marcida* + *P. stenantha*) 1 bp insertion = AATAAAAA (*P. calycina* + *P. parvifolia*) 4 bp insertion = TTTA (*P. hachadoensis* var. *pilosa* + *P. interior* + *P. mendocina* + *P. trivialis*) 8 bp insertion = TTTATTTC (*P. cockaynaiana* + *P. drummondiana* + *P. fax* + *P. porphyrochlados* + *P. pubinervis*) 8 bp insertion = TTTCTATC (both specimens of *P. serpana*) 23 bp insertion = TATATATGAAAGATATAATAAAG (*Dioicopoa s.l.* except *P. holciformis*; *P. pfisteri* + *P. hachadoensis* var. *hachadoensis* + *P. reitzii*) 12 bp insertion = ATTAGAAAAAAT (*P. hachadoensis* var. *pilosa* + *P. interior* + *P. mendocina* + *P. trivialis*) 30 bp deletion (both species of *Aphanelytrum*) 7 bp deletion (*P. fax* + *P. fibrifera* + *P. flabellata* + *P. glauca* + *P. hachadoensis* var. *pilosa* + *P. interior* + *P. marcida* + *P. mendocina* + *P. secunda* + *P. stenantha* + *P. trivialis* + *P. wheelerii*) 5 bp deletion (*P. cucullata* + *P. pauciflora*) 7 bp insertion = ATCAATG (*P. glauca* + *P. hachadoensis* var. *pilosa* + *P. interior* + *P. mendocina* + *P. secunda* + *P. stenantha* + *P. trivialis*) 1 bp insertion = TATTTAA (*P. linearifolia* + *P. spiciformis* var. *ibari*)	

Parsimony analyses were conducted with the four regions analysed separately and combined using TNT (Goloboff *et al.*, 2008) under equal weights. Characters were considered unordered, and uninformative characters were excluded from the analyses. The search strategy consisted of heuristic searches performed using 1000 series of random addition sequences followed by TBR (tree bisection and reconnection) branch rearrangements and retaining ten trees per series. Trees recovered were saved in memory and additionally TBR swapped, retaining a maximum of 100 000 trees. Branches with ambiguous length of 0 or 1 were collapsed, according to collapsing rule 1. A 50 % majority rule consensus tree and a strict consensus tree were generated from the most parsimonious trees. Branch support was assessed using bootstrap analyses (Felsenstein, 1985) with 1000 replicates and heuristic searches of ten series using random taxon entry followed by TBR branch swapping; values given as BS in the text. Incongruence among individual data sets was visually checked by comparing strict consensus trees from individual analyses. When analysing the topology obtained by each partition, all partitions retrieved a similar number of nodes, and most clades were equivalent in species composition. Species that were inconsistent among consensus trees of each individual partition will be reported when presenting results for the combined data set.

Bayesian analysis was conducted for the combined data matrix using MrBayes version 3.1.2 (Ronquist and Huelsenbeck, 2003). jModelTest 2.1.4 (Darriba *et al.*, 2012) was employed to determine the sequence evolution model that best fits the data; ITS and ETS markers were set to TIM2 + G, whereas *trn*T-L and *trn*L-F were set to GTR + G. Models for each partition were selected by the Akaike information criterion (AIC; Akaike, 1974). We carried out two independent runs of 10 000 000 generations using the default priors and four Markov chains (one cold and three heated chains), sampling one tree every 1000 generations. The program Tracer v.1.6 (Rambaut *et al.*, 2014) was used to examine the output parameters from Bayesian analyses to determine stationarity. Trees prior to reaching stationarity were discarded as burn-in, and the remaining trees were used to compute a 50 % majority rule consensus tree and posterior probabilities (shown as BI).

Data on breeding systems in *Poa* were obtained from the literature and personal observations (Table 3; Appendix S1). Species that presented variation in the breeding system (*P. aequigluma*, *P. iridifolia*, *P. matris-occidentalis*, *P. palmeri* and *P. plicata*) were coded as polymorphic (Appendix S1). Although breeding system was not included in the analyses, it was optimized on the majority rule consensus tree of most parsimonious trees using the command ‘common mapping’ by TNT, where the common optimization is represented in the consensus diagram to determine whether breeding system character states were synapomorphies or homoplasies in the clade of interest. To analyse ambiguous optimization on clades of interest, the reconstruction of this character was also evaluated on one of the most parsimonious trees using the command ‘character reconstruction’ by TNT, where all possible optimizations for ambiguous nodes are determined.

**Table 3.** Breeding systems in *Poa* subg. *Poa* supersect. *Homalopoa* in the Americas, with current sectional classification and DNA clade in our study

New World current classification for supersect. *Homalopoa* (+*P. yaganica*)	Species	Clades in our study	Breeding system
***Acutifoliae***	***P. acinaciphylla***	**D + **	Hermaphroditic
	*P. planifolia		Gynodioecious
***Dioicopoa***	***P. alopecurus*** (viviparous sometimes), ***P. arachnifera***, ***P. bergii***, ***P. bonariensis***, ***P. calchaquiensis*, *P. cumingii***, ***P. denudata***, ***P. dolichophylla***, ***P. durifolia***, ***P. holciformis***, ***P. hubbardiana***, ***P. huecu***, ***P. lanigera***, ***P. lanuginosa***,***P. ligularis***, ***P. nubensis***,***P. obvallata***(viviparous sometimes),***P. paposana***, ***P. schizantha***, ***P. spiciformis***,***P. stuckertii***	**D**	Dioecious
	***P. reitzii***	**C (D)**	
	*P. arechavaletae* Parodi, *P. gayana* E. Desv., *P. megalantha* (Parodi) Herter, *P. pedersenii* Nicora, *P. pilcomayensis* Hack., *P. sellowii* Nees, *P. umbrosa Trin., *P. uruguayensis* Parodi		
	*P. iridifolia (viviparous rarely)	**D + **	Gynodiecious/Dioecious
**Unplaced**	***P. spicigera***	**D**	Pistillate
***Madropoa***	***P. pfisteri***	**D + **	Dioecious
	***P. chambersii* (**gd in eastern population)**, *P. cusickii* (**often p, rarely gd)**, *P. douglasii*, *P. fendleriana* (**rarely h, often p)**, *P. macrantha*, *P. piperi*, *P. porsildii***	**C**	
	*P. atropurpurea* Scribn., *P. pringlei* Scribn. (sometimes p), *P. rhizomata* Hitchc. (subdioecious), *P. sierrae* J.T. Howell		
	***P. nervosa*, *P. wheeleri*** (p, rarely with stamens)	**C**	Sequentially Adjusted Gynomonecious
	***P. cuspidata***	**Unplaced**	
	*P. arnowiae* Soreng, *P. diaboli*, *P. tracyi* Vasey		
	*P. confinis* Vasey, *P. leibergii* Scribn., *P. stebbinsii* Soreng (trioecious?)		Gynodioecious
***Anthochloa***	*P. lepidula (sgm?)	**E**	Gynomonoecious
**Unplaced**	***P. ramifera***	**E+, G**	
**Punapoa group**	***P. humillima*, *P. marshallii***		
	*P. anae* Tovar, *P. brevis* Hitchc., *P. denticulata* Hack. (?), *P. dentigluma* Tovar, *P. dissanthelioides*		
	***P. aequigluma*, *P. chamaeclinos*, *P. gymnantha*, *P. perligulata***	**E**	Pistillate
	***P. unispiculata* (d?)**		Gynodioecious
***Dissanthelium s.s.***	***P. calycina* (**h in North America)**, *P. parvifolia*, *P. serpana***	**E + *Dissanthelium***	Gynomonoecious
	*P. arcuata* Refulio, *P. congesta* Refulio, *P. rauhii* Refulio, *P. swallenii* Refulio		
	*P. macusaniensis (E.H.L. Krause) Refulio (gm?, see Sulekic,1999)		Hermaphroditic
**ex *Dissanthelium* (unplaced)**	***P. linearifolia***	**Sister to E**	Gynomonoecious
	*P. thomasii* Refulio, *P. amplivaginata* (Tovar) Refulio		Hermaphroditic
	*P. aequalis (Swallen & Tovar) Refulio		Gynodioecious
	*P. deminuta* Refulio (?), *P. gigantea* (Tovar) Refulio,		Gynomonoecious
	*P. trollii* (Pilg.) Refulio (?)		Dioecious
	*P. boliviana* Refulio		Unknown
***Tovarochloa***	***P. apiculata***	**E + *Tovarochloa***	Hermaphroditic
**Genus *Aphanelytrum***	***A. peruvianum*, *A. procumbens***	**E + **	
***Dasypoa***	***P. laetevirens***, ***P. subspicata***		Gynomonoecious
	*P. parviceps* Hack.		
	***P. scaberula***	**Unplaced**	Hermaphroditic
***Homalopoa s.l.* (incl. sects.*Plicatae*)**	***P. atropidiformis*, *P. matris-occidentalis* (**gm?)**, *P. mulleri*, *P. occidentalis*, *P. plicata*** (h?)		
	*P. aequatoriensis, *P. cucullata, *P. hieronymi*, *P. huancavelicae, *P. pauciflora	**E + **	
	*P. bigelovii* Vasey & Scribn., *P. bolanderi* Vasey, *P. bradei* Pilg., *P. howellii* Vasey & Scribn., *P. jujuyensis*, *P*. *orizabensis* Hitchc., *P. parviceps*, *P. reflexa* Vasey & Scribn., *P. ruprechtii* Peyr. (gm?), *P. seleri* Pilg., *P. strictiramea* Hitchc. (?, stamens often aborted late in development, possibly due to apomixis), *P. tacanae* Swallen, *P. talamancae* R.W. Pohl, *P. wendtii* Soreng & P.M. Peterson (?)		
	***P. bajaensis***	**Sister to F**	
	*P. candamoana, *P. gilgiana, *P. glaberrima, *P. kurtzii*, *P. pearsonii	**F**	Gynomonoecious
	***P. horridula***		Sequentially Adjusted Gynomonecious
	***P. fibrifera***	**E+, G**	
	*P. androgyna* Hack.		
	***P. superata***	**Unplaced**	
	*P. ayacuchensis Tovar (sgm?), *P. chirripoensis R.W. Pohl (sgm or gd?), *P. grisebachii R.E. Fr., *P. leioclada Hack., *P. mucuchachensis Luces, *P. mulalensis Kunth, *P. myriantha* Hack., *P. orthophylla Pilg., *P. oscariana* Negritto & Anton, *P. petrosa Swallen, *P. pilgeri* Negritto & Anton, *P. ragonesei Nicora, *P. scabrivaginata Tovar, *P. soderstromii* Negritto & Anton, *P. trachyphylla Pilg.		Gynomonoecious
	***P. linearifolia***	**Sister to E**	
	***P. lilloi***	**Unplaced**	Gynodioecious
	*P. cabreriana* Anton & Ariza Esp.		
	***P. palmeri* (**trioecious?)	**D + **	Dioecious Gynodioecious
**Sect. *Poa***	*P. yaganica (sgm?)		
**Sect. *Monandropoa***	*P. tucumana* Parodi (1 anther)		Hermaphroditic

Variation and uncertainty in breeding system is given in parentheses: h, hermaphroditic; gm, gynomonoecious (pistillate flowers above perfect flowers in spikelets); sgm, sequentially adjusted gynomonoecious; gd, gynodioecious; d, dioecious; p, pistillate; ?, uncertain.

Breeding system type is from Anton (1978), Anton and Connor (1995), Davidse *et al.* (2010), Giussani (2000), Giussani *et al.* (2012), Negrito and Anton (2000, 2006), Nicora (1978), Parodi (1936), Peterson *et al.* (2006), Refulio-Rodriguez *et al.* (2012), Soreng (1991, 1998, 2007), Soreng and Keil (2003) and Soreng and Peterson (2008).

*New observations (or those differing from the literature).

Species present in the molecular analysis are indicated in bold; for authorship of these species see Appendix S1.

### Molecular dating analyses

To estimate dates, we constructed a matrix based on both nuclear and plastid data following recommendations by Christin *et al.* (2014), who highlighted the importance of using markers from different genomes, considering that nuclear markers are useful complements to plastid markers. We used ITS sequence data as the nuclear partition (ETS was unavailable for most of the outgroup taxa; therefore, it was not considered for this analysis), and *trn*T-L plus *trn*L-F as the plastid regions (hereafter *trn*T-L-F).

We assembled a matrix consisting of 145 terminal taxa of which 96 are species of *Poa*. The species selection represents all lineages of *Poa* supersect. *Homalopoa*; double entries used for phylogenetic analyses were here removed if their positions in the cladogram were confirmed to be close to each other. At least one representative of all New World subgenera of *Poa* was included (Appendix S1).

In the absence of *Poa* fossils to date divergence time among major groups within supersect. *Homalopoa*, we selected appropriate species of the Poaceae family to utilize fossils as calibration points. Outgroup selection comprised representatives of both major Poaceae clades: BOP clade (Bambusoideae, Oryzoideae and Pooideae) and PACMAD (Panicoideae, Aristidoideae, Chloridoideae, Micrairoideae, Arundinoideae and Danthonioideae); taxonomy following Grass Phylogeny Working Group II (2012) and Soreng *et al.* (2015*b*). A particular emphasis was placed on including genera of different tribes of subfamily Pooideae that are closely related to *Poa*.

The topology of the initial tree was constrained to represent relationships among Poaceae as previously reported by Bouchenak-Khelladi *et al.* (2010), and by the Grass Phylogeny Working Group II (2012). Also, phylogenetic relationships within *Poa* supersect. *Homalopoa* were constrained following results of the parsimony/Bayesian analyses (see below).

Fossil evidence, such as micro- and macrofossils, has been used previously to date grasses; this evidence also reflects conflicts among different fossils used as calibration points (Prasad *et al.*, 2005; Strömberg, 2005; Vicentini *et al.*, 2008; Bouchenak-Khelladi *et al.*, 2010; Christin *et al.*, 2014). To take into account different dates proposed for a particular clade, we used a log-normal prior distribution on nodes constraining dates to a minimum age represented by a well-known fossil for the group (offset value) and setting the upper bound (95 %) on a secondary calibration point based on the earliest fossil proposed for the same clade; standard deviation was set to 1.

We calibrated six external nodes for this data set. (1) We used a multiflowered-spikelet fossil which shares characters of both the BOP and PACMAD clades to calibrate the most external node of the Poaceae at 55 million years ago (Mya) (Crepet and Feldman, 1991); also, the date accords with a fossil leaf impression with bambusoid affinities from the Eocene (Frenguelli and Parodi, 1941; Brea and Zucol, 2007). Hence, the offset was set at 55 Mya ranging to 70 Mya (upper bound, 95 %) based on the grass-like pollen fossil described in Linder (1986). (2) Then, divergence time of the Pooideae clade from the PACMAD clade was set at a minimum age of 42 Mya estimated from phytolith grass fossils described by Zucol *et al.* (2010). Phytoliths were found in sediments of the Gran Barranca member of the Sarmiento Formation in Argentina representing festucoid and pooid morphotypes; similarly, phytoliths were also found in fossil dung beetle brood balls (Coprinisphaera) from the Middle Eocene–Early Miocene Sarmiento Formation by Sánchez *et al.* (2010). The upper bound (95 %) was set at 67 Mya based on phytolith fossils of dinosaur coprolites from India as stated in Prasad (2005). (3) For the PACMAD clade, a minimum age was estimated at 31 Mya based on phytoliths from the Gran Barranca (Zucol *et al.*, 2010), while the upper bound was set at 52 Mya. (4) Based on phytolith evidence, the offset for the Chloridoids was 14 Mya (Strömberg, 2005) and the upper bound was set at 19 Mya based on Dugas and Retallack (1993). (5) Based on a well conserved stipoid fossil, the silicified reproductive bracts of *Berriochloa* (Thomasson, 2005), we set the minimum age at 7·6 Mya for the Stipeae node, ranging to 34 Mya (95 %) based on another fossil with stipoid similarities (Prasad *et al.*, 2011). (6) To calibrate internal branches within Panicoids, we used a fossil related to *Setaria* to set a minimum age of 7 Mya (Elias, 1942) for *Panicum* as both genera are closely related (Giussani *et al.*, 2001).

We used a relaxed Bayesian molecular clock method to consider variation in evolutionary rates across the phylogeny (Drummond *et al.*, 2006). The analysis was set with prior on the distribution of node ages approximated by a Yule speciation process and evolutionary rates among branches followed a log-normal distribution and considered uncorrelated as implemented in BEAST v 1·8 (Drummond *et al.*, 2002–2013). The substitution models used and the Bayesian analysis were implemented as reported in ‘Phylogenetic analyses’.

### Geographic data

Data from herbarium databases of the Darwinion Institute (Documenta Florae Australis: http://www.darwin.edu.ar/iris/), Smithsonian Institution, Canadian Museum of Nature and Negritto’s database were used to score 2786 specimens of the American species of *Poa* supersect. *Homalopoa* for geographical distribution (Supplementary Data Appendix S2). Geographic co-ordinates were obtained from specimen labels or georeferenced localities; the package ‘raster’ (Hijmans and van Etten, 2015) available in the R statistical package 3·2·2 (R Development Core Team, 2010) was employed to plot the specimens. Altitude as represented in the map has been obtained from WorldClim–Global Climate Data (http://www.worldclim.org/).

## RESULTS

### Phylogenetic analyses

A total of 124 specimens were considered for the phylogenetic analyses; only two species (*P. pfisteri* and *P. schizantha*) were difficult to amplify and discarded for the ITS data set. Characteristics of individual partitions (*trn*T-F, *trn*L-F, ETS and ITS) are presented in Table 2; informative indels are reported and discussed if they have phylogenetic significance. The aligned ETS partition yielded a total of 560 bp; the ITS was 685 bp long, while the plastid *trn*T-L-F region consisted of 2204 bp.

The combined matrix of nuclear and plastid markers is 3449 bp long and 417 characters are phylogenetically informative (Table 2). Trees obtained from parsimony and Bayesian inference analyses are presented in Supplementary Data Figs S1 and S2, respectively. Similar clades were obtained in both analyses.

Subgenus *Poa* resolved as monophyletic (BS = 100; BI = 1). Both varieties of *P. hachadoensis* and *P. mendocina* were united in a clade (X-clade) that is sister to a group with both supersections of the subgenus *Poa*: supersect. *Poa* (BS = 100; BI = 1) and supersect. *Homalopoa*. However, some discrepancies were found among partitions: the ETS partition showed *P. pratensis* subsp*. alpigena* and *P. sibirica* included within the *Homalopoa* clade, while in the *trn*T-L-F and ITS consensus trees *Homalopoa* was not recovered.

Supersection *Homalopoa* is monophyletic and supported when the data sets are combined (BS = 98; BI = 0·8). Within this group, major clades and species are resolved in a basal trichotomy: *Poa chaixii* Vill.; the New World *Homalopoa* species, clade A (BS = 89; BI = 0·94); and all species of the Australasian sect. *Brizoides*, clade B (BS = 98; BI = 1). Relationships among major clades within the New World *Homalopoa* clade collapse in a polytomy in the strict consensus parsimony tree (Fig. S1) and in the Bayesian tree (Fig. S2).

Most of the dioecious species of North America are united in the monophyletic sect. *Madropoa*, clade C (BS = 83; BI = 0·99), with the Brazilian *P. reitzii* Swallen, previously classified in sect. *Dioicopoa*, in a basal position within clade C. The sect. *Dioicopoa* is monophyletic (clade D, sect. *Dioicopoa s.s.*: BS = 99; BI = 1) if including all South American dioecious species, except for *P. iridifolia* Hauman and *P. pfisteri* Soreng (ITS data lacking), plus the North American dioecious species *P. arachnifera.* Clade D also includes *P. spicigera*, a species previously unplaced. The broader moderately supported D+ clade (BS = 73; BI = 1) includes section *Dioicopoa s.s.* plus other gynodioecious, dioecious and hermaphrodite species (*Dioicopoa s.l.*). Members of this clade share a 23 bp insertion of the *trn*T-L region (TATATATGAAAGATATAATAAAG) that is a duplication of the previous segment (Table 2); it is absent only in *P. bonariensis, P. holciformis* and *P. pfisteri*, and present in *P. reitzii* and *P. hachadoensis*). *Poa planifolia* Kuntze, a gynodioecious species classified in sect. *Acutifoliae* from South America (Appendix S1), was resolved as sister taxon to sect. *Dioicopoa s.s.* Other taxa resolved as members of D+ are: *P. iridifolia* (classified in sect. *Dioicopoa*; dioecious and gynodioecious, pers. obs.) of the Argentine Pampas hills; *P. palmeri* Soreng & P.M. Peterson from Mexico (sect. *Homalopoa s.l*.; partially dioecious, because of the presence of hermaphrodite, pistillate and staminate individuals); *P. yaganica* Speg., a southern Patagonian species (subg. *Poa* supersect. *Poa*; gynodioecious); and *P. acinaciphylla* E. Desv. (sect. *Acutifoliae*; hermaphroditic) from the central Andes of Argentina and Chile. The position of *P. reitzii* in clade C needs to be corroborated; although resolved with sect. *Madropoa* in the combined analysis and in both individual phylogenies from ETS and *trn*T-L-F, it was included within clade D, sect. *Dioicopoa s.s.*, in the ITS parsimony majority rule tree, but was unresolved in the strict consensus tree.

The following groups were supported (all from South America unless noted). The Punapoa group was not monophyletic, but we detected one core group of dwarf species that inhabit the Andean Altiplano and form a highly supported sub-clade E (BS = 100; BI = 1; Punapoa p.p. 1). An extended clade E+ includes species of sect. *Dissanthelium* (BI = 0·78), both species of *Aphanelytrum* together with *P. apiculata* Refulio of sect. *Tovarochloa* (BS = 100; BI = 1), several species of *Homalopoa s.l*. and two species of sect. *Dasypoa*. A group of four dissimilar species are included in subclade G (BS = 62; BI = 0·85): *P. humillima* Pilg. and *P. marshallii* Tovar (Punapoa p.p. 2), and *P. fibrifera* Pilg. and *P. ramifera* Soreng & P.M. Peterson. A separate group of South American *Homalopoa s.l.* species (*P. candamoana* Pilg., *P. gilgiana* Pilg., *P. glaberrima* Tovar, *P. horridula* Pilg., *P. kurtzii* R.E. Fr. and *P. pearsonii* Reeder) are included in a highly supported clade F (BS = 99; BI = 1), with *P. bajaensis* Soreng (from Mexico) resolved as sister. *Poa gymnantha* Pilg., classified with the core Punapoa group (above), was included unambiguously in subclade E by the ETS, ITS and the combined data set analyses.

### Evolution of breeding systems

**Fig. 3.**
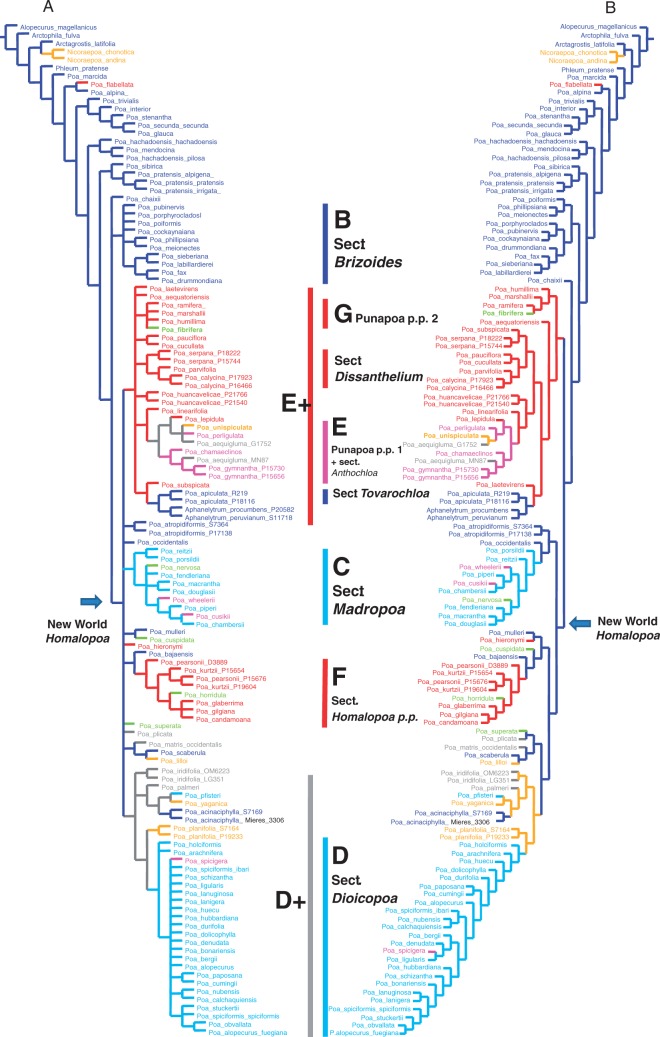
Optimization of the breeding system. (A) Majority rule consensus tree from the most parsimonious trees optimized by ‘common mapping’ in TNT; (B) character reconstruction in one or the most parsimonious trees. Bars and letters indicate principal clades within *Poa* supersect. *Homalopoa* as indicated by our results. Colours represent variation of the breeding system among species and follow references as presented in Fig. 2; grey colour represents ambiguous optimization on branches.

**Fig. 4.**
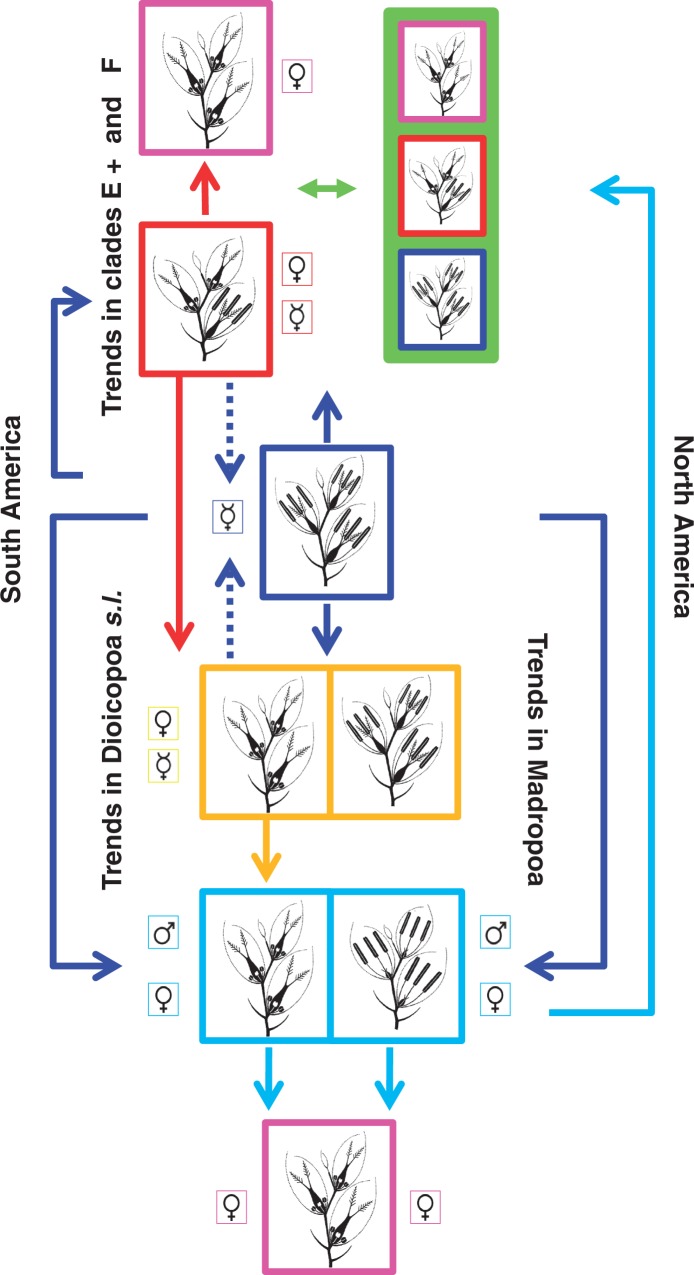
Evolutionary pathways of the breeding systems in the New World *Homalopoa*. Colours represent variation of the breeding system among species and follow references as presented in Fig. 2. Principal trends among American clades are shown, with arrows as explained in the text.

When optimizing the reproductive system onto the most parsimonious trees, hermaphroditism appears as the ancestral state for *Poa*. Since basal relationships within the New World *Homalopoa* clade were unresolved, different solutions to the evolution of the reproductive system are possible (Fig. 3). However, ancestral states for each major clade were recovered unambiguously for one reproductive system as seen when optimizing in the majority rule consensus tree (Fig. 3A). Based on possible optimizations of the basal node of the New World *Homalopoa* clade (there are 1504 possible resolutions for the polytomy), dioecy appeared at least once, if *Madropoa* (M) and D+ are closely related (an equally parsimonious relationship, although not supported in parsimony analyses), or twice if those clades had independent origins within the New World supersect. *Homalopoa.* Gynomonoecy, although present in most species of E+ and F clades, is also found in a single species (*P. hieronymi* Hack.) as a derived state from hermaphrodite ancestors; and a reversal to hermaphroditism appears to have occurred in section *Tovarochloa* (where there is only one flower per spikelet) plus *Aphanelytrum*. At least two different reconstructions were possible for the *Dioicopoa s.l.* (clade D+), with dioecy or gynodioecy being equally parsimonious ancestral states. For sub-clade E, both pistillate and gynomonoecy are possible states for the ancestor of this monophyletic group. Gynodioecy appeared three to four times in *Homalopoa* clade A: as an ancestral state of *Dioicopoa s.l*., with a reversal to hermaphroditsm in *P. acinaciphylla*, or twice within *Dioicopoa s.l.*; in *P. unispiculata* (sub-clade E); and in *P. lilloi* from the Altiplano. A diagram with possible evolutionary pathways of the breeding systems in the New World *Homalopoa* is presented in Fig. 4.

### Geographic distribution

**Fig. 5.**
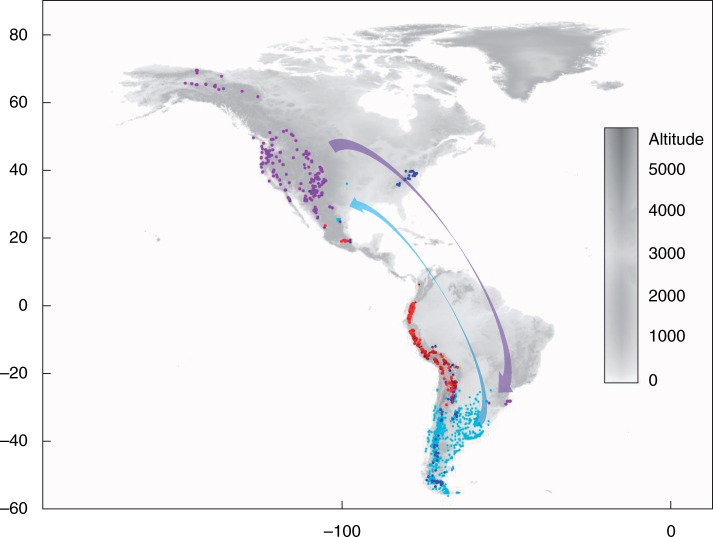
Current distribution of New World species of *Poa* supersect. *Homalopoa*. Colours indicate species as grouped by the phylogenetic results. Light blue, clade D + (sect. *Dioicopoa s.l.*); violet, clade C (sect. *Madropoa*); red, clade E+; red circle with black border, clade F; blue, species not grouped. Arrows represent long-distance dispersal events which occurred in the past: the light blue arrow shows two long-distance dispersal events from the South American D+ clade to North America (*P. arachnifera* and *P. palmeri*); the violet arrow shows a long-distance dispersal event from the North American clade C to South America (*P. reitzii*). Specimen coordinates are presented in Appendix S2.

A geographic distribution map was obtained for the American species of *Poa* supersect. *Homalopoa* using georeferrenced specimen data (Fig. 5). Major clades were visualized onto the map and showed a particular distribution associated with restricted areas. Dioecy according to phylogenetic results appeared twice in clades C and D both in North and South America, respectively; only *P. reitzii* and *P. arachnifera* have a different distribution according to their phylogenetic affiliations. Gynomonoecious species (clades E+ and F) are clearly distributed along the Andes in northern Argentina and Chile to Colombia in South America and in a few localities in Mexico.

### Molecular dating: time of divergence in *Poa*

**Fig. 6.**
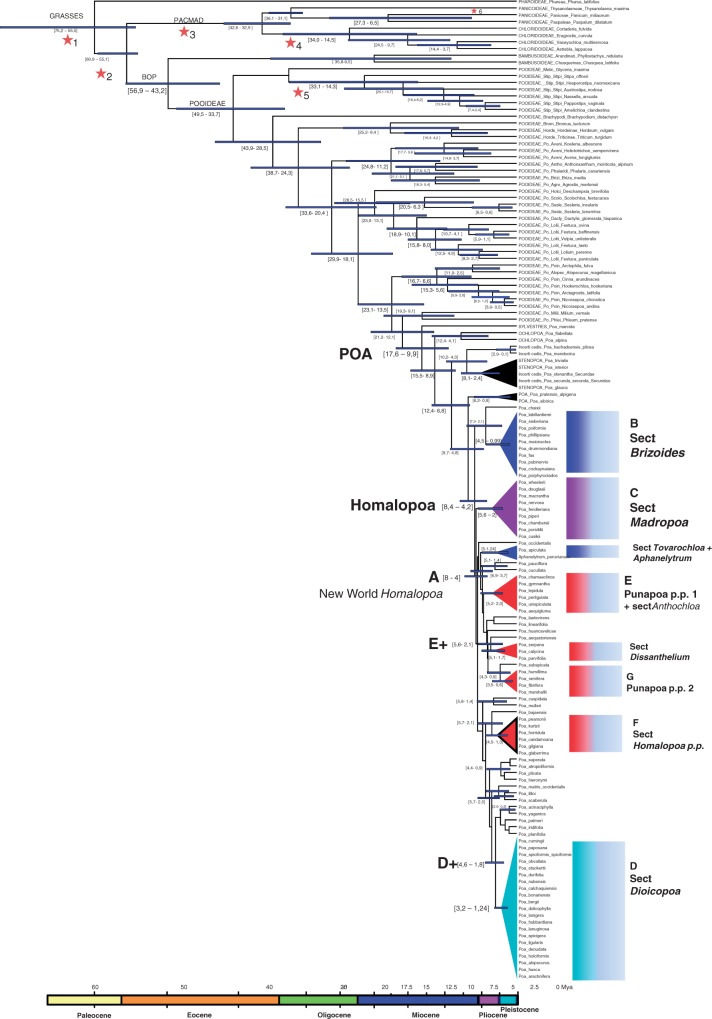
*Poa* chronogram based on a relaxed Bayesian clock. Node bars represent 95 % confidence intervals of divergence times. Stars and numbers indicate dated nodes based on fossil evidence as described in the Materials and Methods. Letters indicate principal clades within *Poa* supersect. *Homalopoa* as shown by our results; traditional sectional treatments are also presented as introduced in the text and Table 1. Colours indicate major groupings: light blue, dioecious species in South America + *P. arachnifera* (sect. *Dioicopoa*) + *P. spicigera*; violet, dioecious species in North America (sect. *Madropoa*) + *P. reitzii*; red, gynomonoecious species of clade E+; red triangle with black border, gynomonoecious species of clade F; blue, hermaphroditic species of sect. *Brizoides* and sect. *Tovarochloa* and *Aphanelytrum*.

The estimated ages for crown and stem nodes are shown in Fig. 6. Divergence dates for both major clades BOP and PACMAD were estimated at 61–55 Mya and yielded similar results to those previously reported (Vicentini *et al.*, 2008; Bouchenak-Khelladi *et al.*, 2010; Wu and Ge, 2012). The crown age of PACMAD was estimated at 43–33 Mya, while the age of the BOP was estimated at 57–43 Mya, agreeing with previous estimations of a radiation in the Middle Eocene to Early Oligocene (Zachos *et al.*, 2001; Strömberg 2005; Inda *et al.*, 2008; Bouchenak-Khelladi *et al.*, 2010; Edwards *et al.*, 2010; Wu and Ge, 2012).

Within the subfamily Pooideae, the crown radiated 50–34 Mya ago and, within this clade, Stipeae diverged between 33 and 14 Mya. Because our major concern is with *Poa*, results for other groups are presented in Fig. 6 and were checked for constancy with previous dating.

The estimated age for the crown node of the genus *Poa* is about 18–10 Mya, in the Middle Miocene, while the supersection *Homalopoa*, although few representatives were included from the Old World, is estimated at about 8·4–4·2 Mya. Radiation of species into the Americas is estimated at almost the same age, in the Late Miocene–Early Pliocene (8–4 Mya). Within the New World *Homalopoa* clade, a polytomy is shown for basal lineages; major clades diverged at similar ages or periods, mostly in the Pliocene to Early Pleistocene, about 5–2 Mya (Fig. 6). Section *Dioicopoa s.s.* (clade D) evolved during the Late Pliocene to Early Pleistocene in southern South America, and is one of the most recently evolved lineages within *Homalopoa* (3·2–1·24 Mya). Section *Madropoa* in North America is estimated to be older (5·6–2 Mya) than *Dioicopoa s.s*. Results for other lineages within *Homalopoa* are presented in Fig. 6.

## DISCUSSION

Monophyly of *Poa* has been extensively discussed, and resolution of phylogenetic relationships among closely related genera has helped to elucidate the identity of *Poa* (Soreng *et al.*, 1990, 2015*a*; Gillespie and Soreng, 2005; Gillespie *et al.*, 2007, 2008, 2009, 2010; Refulio-Rodriguez *et al.*, 2012). In our analyses, *Poa* was resolved as monophyletic, and representatives of the major subgenera were included to anchor relationships among sections of supersect. *Homalopoa*. Despite high morphological and taxonomic diversity (Fig. 1; Table 1), molecular studies have had little success in resolving relationships within supersect. *Homalopoa* (Gillespie and Soreng, 2005; Gillespie *et al.*, 2007, 2009; Refulio-Rodriguez *et al.*, 2012). In our work, all American species were recovered in clade A, named the New World *Homalopoa* clade (Figs S1 and S2). Clade A, the Eurasian *Poa chaixii* and the Australasian sect. *Brizoides* together comprise the *Poa* supersect. *Homalopoa* clade. Section *Brizoides* was monophyletic, in agreement with Gillespie *et al.* (2009) and Refulio-Rodriguez *et al.* (2012). In their study of Australasian *Poa*, Birch *et al.* (2014) greatly increased sampling in sect. *Brizoides* and resolved three sub-clades, but did not include *Homalopoa* species from other sections or from outside the region. Our study is the first to provide significant and meaningful structure among New World *Homalopoa*. Although basal relationships were poorly resolved, seven clades (described in detail below) were resolved that correspond well to the sectional classification and/or to morphological characteristics. Four of these clades (sects. *Dioicopoa s.s.*, *Dissanthelium s.s.*, *Madropoa* and *Tovarochloa* + *Aphanelytrum*) were also detected in previous studies, but mostly with less support or fewer species sampled (Soreng, 1990; Gillespie *et al.*, 2008, 2009; Refulio-Rodriguez *et al.*, 2012).

The evolutionary history of *Poa* in the Americas is linked to the diversification of breeding systems in the region. While hermaphroditism was revealed as plesiomorphic for the genus *Poa* and for supersect. *Homalopoa*, variation in reproductive systems occurred principally within the New World species of supersect. *Homalopoa* (Fig. 3; Table 3). Reversals to hermaphroditism occur in the *Tovarochloa* + *Aphanelytrum* clade, which is embedded within the expanded gynomonoecious clade E+. Reversion to monoecy has also been reported in several groups (Schaefer and Renner, 2010; Ming *et al.*, 2011), and it is also possible in *Poa*. Other hermaphroditic species such as *Poa atropidiformis* Hack., *P. mulleri* Swallen, *P. occidentalis* and *P. scaberula* are part of a basal polytomy and have retained the plesiomorphic state. *Poa bajaensis*, sister to the gynomonoecious clade F, also retains the plesiomorphic state. The hermaphrodite *P. acinaciphylla* may represent a link to the ancestor of the D+ clade or a reversal to hermaphroditism within the D+ clade. Here we review the breeding systems and groups detected in our study.

### Dioecy–gynodioecy

Dioecy appeared three times in *Poa*. Two origins of dioecy occurred within supersect. *Homalopoa*, evolving independently in the monophyletic sections *Dioicopoa* and *Madropoa*; however, when resolving the major polytomy within the New World *Homalopoa*, a common ancestor between D+ and *Madropoa* cannot be ruled out. Another event involves three unclassified dioecious New Zealand species [*P. subvestita* (Hack.) Edgar, *P. sudicola* Edgar and *P. foliosa* (Hook. f.) Hook. f.; not sampled here] which, along with 13 New Zealand and eight New Guinea hermaphroditic species (the latter to which the name *Poa* sect. *Pauciflorae* Pilg. ex Potztal applies), align in the nrDNA X-clade and appear to have a reticulate origin (Gillespie *et al.*, 2009; Birch *et al.*, 2014).

In South America, *P. acinaciphylla*, a hermaphroditic species of Chile and Argentina, appears to be closely related to a group of gynodioecious and dioecious species that together constitute the *Dioicopoa s.l.* clade (D+): *P. iridifolia*, *P. palmeri*, *P. planifolia, P. pfisteri*, *P. yaganica* and the *Dioicopoa s.s.* clade (Table 3; Figs S1 and S2). *Poa planifolia* is a gynodioecious species that occurs in the same general area as *P. acinaciphylla* in the Andean region between latitude 32 ° and 34 °S in Argentina and Chile (both placed in sect. *Acutifoliae*). Soreng and Peterson (2008) suggested a hybrid origin for *P. acinaciphylla* involving *P. planifolia* and *P. holciformis* of sect. *Dioicopoa s.s*. Our data do not support this hypothesis, although these species are related by ancestral relationships: *P. planifolia* is sister to the D clade (where *P. holciformis* is included), and *P. acinaciphylla* and *P. planifolia* are included in the D+ clade by plastid data (not resolved by the nrDNA data), showing a close relationship to *Dioicopoa s.s.* Although Soreng and Peterson (2008) suggested that *P. pfisteri* might be related to *P. diaboli* of sect. *Madropoa*, and *P. yaganica* to *P. pratensis* of supersect. *Poa* sect*. Poa*, we here confirm their relationship to *Dioicopoa s.l*. *Poa palmeri*, an endemic species from the Sierra Madre Oriental in Mexico, is here linked to *Dioicopoa s.l.*; specimens of this species are most frequently hermaphroditic (although anthers are sometimes aborted late in development), but some plants are completely staminate or pistillate (tentatively identified as trioecious by Soreng and Peterson, 2012), suggestive of a possible step toward dioecy. *Poa palmeri* and *P. arachnifera* are the only two taxa included in the D+ clade that are found in North America (Fig. 5). Relationships of both taxa to the South American dioecious clade are probably the result of separate long-distance dispersal events in the past taking place from South to North America. Although the two could be relicts from earlier diversification in North America, *P. arachnifera* is fully dioecious, with sexual dimorphism and panicles fully developed in the same way as most South American sect. *Dioicopoa* species, thus supporting more recent long-distance dispersal northward from South America.

### Dioicopoa s.s.

Section *Dioicopoa* as previously circumscribed included 30 South American species and *P. arachnifera* from North America (Parodi, 1936; Rosengurtt *et al.*, 1970; Anton, 1978; Nicora, 1978; Smith *et al.*, 1981; Longhi-Wagner, 1987; Giussani, 2000; Soreng *et al.*, 2003; Giussani *et al.*, 2012), 23 (80 %) of which were included in the analyses (Table 3). The majority of these sampled species resolved in clade D (21 species), a group which we define here as sect. *Dioicopoa s.s*. All species of clade D are dioecious except for a Peruvian unplaced species *P. spicigera* for which only pistillate individuals are known (Tovar, 1993; Giussani, 2000; Giussani *et al.*, 2012). The latter species would be the only strictly pistillate species of the group, having changed to apomictic seed reproduction in *Dioicopoa s.s*. However, its morphology suggests a relationship to *P. perligulata* of sub-clade E, hinting at a possible deeper reticulate origin, a hypothesis that needs to be tested.

*Dioicopoa s.s.* species are adapted to some of the driest and coldest areas in South America, and many of its species occur in Patagonia of Argentina and Chile; some species also inhabit temperate regions in the Pampas in Argentina, Brazil, Paraguay and Uruguay, and the equivalent habitat in the southern Great Plains of North America (*P. arachnifera*), or high elevations of the ‘Altiplano’ in Argentina (Giussani *et al.*, 2008), Bolivia, Chile and Peru (Fig. 5), or coastal fog belts and sand dunes in arid central Chile.

Surprisingly, *P. iridifolia*, an endemic species from the Ventania and Tandilia mountain ranges in southern Buenos Aires province of Argentina (Torres, 1970) and considered a member of the *P. dolichophylla* complex of sect. *Dioicopoa* (Giussani, 2000), is apart from *Dioicopoa s.s.* This result, together with new observations by Villamil and Giussani that confirm that dioecy is not completely established in its populations (since this species presents hermaphroditic and pistillate individuals, or, similarly to *P. palmeri*, hermaphroditic, staminate and pistillate phases), suggests that it does not belong to *Dioicopoa s.s.* but rather to a group of diclinous species that resolved at the base of the D+ clade (described above).

A synapomorphic state for *Dioicopoa s.s.* (excluding *P. spicigera*) is the highly specialized sexual dimorphism. Characters linked to sexes are: the copious hairiness in the pistillate floret (hairs along the nerves and/or internerves of lemmas, and usually woolly or plicate hairs in three well-developed tufts on the callus), with the exceptions of glabrous individuals of *P. bergii*, and three glabrous species: *P. holciformis*, *P. huecu* and *P. nubensis*; plant size (pistillate plants taller than staminate plants); and quantitative traits of the spikelet (pistillate larger than staminate, and often fewer flowered) (Giussani, 2000). Dimorphism is also related to spatial segregation of sexes adapted to differences in microsite quality and resource availability (Bertiller *et al.*, 2000, 2002), or to grazing pressure (Graff *et al.*, 2013), revealing a high degree of specialization for dispersal and habitat segregation within the section. As a result*, Dioicopoa s.s.* is remarkably variable in morphology, distribution and habitats. In addition to sexual dimorphism, species are characterized by contracted and linear or elliptic (less often open-pyramidal) panicles, spikelets with three or more florets, and staminate flowers with long fertile anthers [1·5–3·5 (–4·5) mm long]. Plants mainly grow below 3500 m elevation (only *P. calchaquiensis* and *P. nubensis* grow above this altitude; Giussani *et al.*, 2008). It is evident that speciation resulted in morphological discontinuities more rapidly than fixation of molecular variation in the DNA markers studied; hence few monophyletic groups are detected within this section in the molecular phylogeny. Several far southern species exhibit pseudoviviparous reproduction in addition to sexual reproduction (Moore and Doggett, 1976).

### Madropoa

This section evidently originated in North America and has a centre of diversity in the mountains of the western USA, with the highest species diversity and numerous endemics in California, Oregon and Washington (Fig. 5). *Madropoa s.s*. (Soreng, 1991) originally included 11 species and one nothospecies of western North America, six of which are dioecious, three are gynodioecious, one trioecious, one with gynodioecious and dioecious populations, and the nothospecies is predominantly pistillate–apomictic. Most of the taxa have folded involute-margined leaf blades that adaxially are usually scabrous or coarsely puberulent on and between the veins, contracted panicles, and occur in open habitats. Eight species of the *P. nervosa* complex were added (Soreng, 2007) to *Madropoa* along with a new species, *P. diaboli* Soreng & D.J. Keil (Soreng and Keil, 2003): five are sequentially gynomonoecious (Soreng and Keil, 2003), one has gynodioecious and dioecious populations, one is sub-dioecious, one is dioecious and one is pistillate–apomictic. These mostly have flat or folded leaf blades that adaxially are smooth and glabrous between the veins (except the pistillate–apomict), most have open panicles, and they are generally confined to forested habitats. Callus hairs in *Madropoa* may be absent, arise in a single dorsal tuft or be a bit diffuse or arranged as a crown around the callus, but none occurs in three isolated tufts as in *Dioicopoa*. All 21 *Madropoa* species are diclinous (Soreng, 2007), but none exhibits simple gynomonoecy. Of these, five have numerous pistillate–apomictic plants, and apomixis occurs in over half or all of their geographic ranges (Table 3). The complexity of breeding systems in *Madropoa* is well illustrated by the four subspecies of *P. cusickii* Vasey, two of which are sexually reproducing, gynodioecious and dioecious, or strictly dioecious (subspp. *cusickii* and *pallida* Soreng, respectively), both with frequent populations that are strictly pistillate–apomictic in more arid and colder habitats, and two subspecies which are strictly pistillate–apomictic occurring in sub-alpine and low alpine habitats [subspp. *epilis* (Scribn.) W.A. Weber and *purpurascens* (Vasey) Soreng] (Soreng, 1991). Sequentially adjusted gynomonoecy is less frequent than dioecy among species of sect. *Madropoa* (Table 3), occurring mostly in the *P. nervosa* complex; only *P. nervosa* is present in our analyses and included in clade C. *Poa cuspidata*, although sequentially gynomonoecious and included in the *P. nervosa* complex of sect. *Madropoa* by Soreng (1991, 2007), is not resolved within this clade, but instead is linked to *P. mulleri* (hermaphroditic), both in a basal polytomy within the New World *Homalopoa* clade*.* The phylogenetic position of other species of sect. *Madropoa* exhibiting sequentially adjusted gynomonoecy, such as *P. arnowiae* Soreng, *P. diaboli* and *P. tracyi* (Soreng, 2007), still needs to be determined (Table 3). *Poa wheeleri* Vasey, a high polyploid apomictic species that is pistillate with vestigial (or very rarely developed) anthers, also belongs in clade C; it was suggested to be a hybrid between *P. cusickii* and a member of the *P. nervosa* complex (Soreng, 2007) not in our study.

Surprisingly, a Brazilian endemic, *P. reitzii*, is included in the North American *Madropoa* clade with high support in the ETS and *trn*T-L-F trees, and with moderate support in the combined trees. If this relationship is later confirmed, it would imply that an early long-distance dispersal event of an ancestral species took place between North and South America, followed by isolation and speciation, with the southern descendant, *P. reitzii*, now restricted to high elevations of the Serra Geral in southern Brazil (Fig. 5). However, more samples of this species are needed to confirm this relationship. Its breeding system is thought to be fully dioecious (Smith *et al.*, 1981). Morphologically it seems close to *P. iridifolia*, with its strongly compressed and wing-keeled sheaths, broad, flat, somewhat firm blades, large loose panicles, presence of three tufts of callus hairs on each pistillate floret, and glabrous staminate florets (a dimorphism characteristic of *Dioicopoa s.s*.). In contrast to *Dioicopoa*, sexes of sect. *Madropoa* are not dimorphic or are only obscurely so, as described for *P. fendleriana* (Steud.) Vasey by Soreng (1985).

### Gynomonoecy and exclusively pistillate species

Simple gynomonoecy is a widespread breeding system among species of *Poa*, where it is readily diagnosed by spikelets with the upper one (or two) flower pistillate and the lower one (or two) perfect in all individuals. It is well expressed and fixed in at least 40 Andean species of South America (Negritto, 1998; R. J. Soreng, pers. obs.), but only tentatively diagnosed in three species endemic to North America (Anton and Connor, 1995; Soreng and Peterson, 2012); Fig. 5. The frequency of simple gynomonoecism has been found to be six times greater in South America than in the Old World (Anton and Connor, 1995); in the Old World it occurs in about 26 species from Eurasia, Africa and New Zealand, of which only four are confirmed to belong in supersect. *Homalopoa* (Anton and Connor, 1995; Edgar and Connor, 2010; R. J. Soreng, pers., obs.). It was first identified in *P. annua* (Hackel, 1904), and later in its mainly Eurasian parents, *P. infirma* Kunth and *P. supina* Schrad. (Soreng *et al.*, 2010). Simple gynomonoecy appears in clades E+ and F (Fig. 3); within E+ there appears to be a reversal to hermaphroditism in sect. *Tovarochloa* + *Aphanelytrum.*

Sequentially adjusted gynomonoecy diagnoses require studying populations through time (Soreng and Keil, 2003; earlier identified as ‘partial gynodioecy’ by Soreng and Hatch (1983) and Soreng (1985), and its nature confused by Anton and Connor, 1995). This breeding system is known or estimated for five or six species from North America (best studied in the *P. nervosa* complex) and 5–7 species of South America (where it has been tentatively diagnosed both by R. J. Soreng, pers. obs., and M. A. Negritto, pers. obs.). Sequentially adjusted gynomonoecious species are scattered in our trees, appearing to have evolved multiple times within New World *Homalopoa*: *P. nervosa* (Clade C, sect. *Madropoa*), *P. fibrifera* (sub-clade G), *P. horridula* (clade F), *P. cuspidata* and *P. superata* (both occur in unresolved clades) and *P. palmeri* (possibly trioecious, clade D+); *P. horridula* and *P. fibrifera* appear to be derived from simple gynomonoecious species. The diverse placements of putative sequentially adjusted gynomonoecious species suggest that this breeding system may represent a transitional state in derived species. It is remarkable that sequentially adjusted gynomonoecy is also expressed in seven Asian species (Zhu *et al.*, 2006; R. J. Soreng, pers. obs.).

In our study, two major clades (clades E+ and F, Figs S1 and S2) of simple gynomonoecious species of the Altoandina and Puneña phytogeographical provinces [including the highest elevations all along the Andes, the former province above 4200 m a.s.l. and the latter from 3200 to 4400 m (Cabrera and Willink, 1980)] are recovered in the phylogeny. The number of times simple gynomonoecy originated depends on the resolution of basal clades, varying from two to four depending upon optimizations (Fig. 3).

### Clade F

This is a group of gynomonoecious species from the Andes, ranging from Colombia (*P. horridula*) to northern Argentina and Chile (Fig. 5). All these species inhabit prairies on slopes at high elevations (3000–4800 m). This group is morphologically coherent, characterized by medium to large plants (30–150 cm), with panicles ovate to pyramidal, open or loosely contracted, lax, with spikelets mostly concentrated at the medial or apical portion of branches. Two groups of species are evident in Figs S1 and S2. *Poa kurtzii* and *P. pearsonii* are both densely cespitose, xeromorphic species of southern distribution frequent at medium to high elevations in arid Argentina and Bolivia. *Poa candamoana*, *P. gilgiana*, *P. glaberrima* and *P. horridula* are mesomorphic, weakly rhizomatous plants (only *P. candamoana* is cespitose), mostly distributed from Bolivia to Ecuador; *P. horridula* is the most widely distributed species (Argentina to Colombia) with the largest and most robust plants (up to 150 cm high). All species have a simple gynomonoecious breeding system, except for *P. horridula*, which is sequentially adjusted gynomonoecious (R. J. Soreng, pers. obs.), or possibly gynodioecious (Negritto, 1998). A possible connection to a hermaphroditic ancestor is based on the sister relationship of the Andean clade F with the hermaphrodite *P. bajaensis* (although with low probability), a Mexican endemic from Sierra San Pedro Mártir in Baja California. *Poa bajaensis* grows at medium to high elevations and also presents similar open, lax panicles, with basally naked branches.

### Clade E+ 

This clade unites central and northern Andean species of South America, mostly concentrated at higher elevations of northernmost Argentina, Bolivia, Ecuador and Peru; only *P. chamaeclinos* and *P. gymnantha* (both pistillate–apomicts), and a hermaphroditic form of *P. calycina* (normally simple gynomonoecious) reach Mexico in North America (Fig. 5). Four sub-clades are well supported within E+: sect. *Dissanthelium*, sect. *Tovarochloa* + *Aphanelytrum*, sub-clade E and sub-clade G (Figs S1 and S2). The 12 species placed in the informal group Punapoa (by Soreng *et al.*, 2003), plus *P. unispiculata* (Davidse *et al.*, 2010), are divided between sub-clades G and E. We consider Punapoa to be an unnatural group in both its initial (Soreng, 1990) and later sense (Soreng *et al.*, 2003; Davidse *et al.*, 2010). Section *Dissanthelium s.s.*, recently recircumscribed by Refulio-Rodriguez *et al.* (2012), includes eight simple gynomonoecious species and one apparently hermaphroditic species [*P. macusaniensis* (E.H.L. Krause) Refulio], distributed from Mexico to Argentina; species are cespitose and dwarf, with two-flowered spikelets (upper floret pistillate), glumes equal to or longer than the florets, and anthers 0·2–1·1 mm long. The second sub-clade comprises the monotypic section *Tovarochloa* (with the single species *P. apiculata*) and *Aphanelytrum*, which was also supported in the analyses of Gillespie *et al.* (2008) and Refulio-Rodriguez *et al.* (2012). All members are hermaphroditic; if simple gynomonoecism was fixed in the ancestor of clade E+, as appears likely, then this sub-clade represents a reversal to hermaphroditism. *Poa apiculata* is a rare dwarf annual species with one flower per spikelet that inhabits shallow ephemeral pools at high altitudes (Refulio-Rodriguez *et al.*, 2012). Thus, a reversal to hermaphroditism, at least for sect. *Tovarochloa*, could be explained by the loss or suppression of the terminal pistillate floret of a simple gynomonoecious ancestor. *Aphanelytrum* is a genus currently with two species (Sánchez Vega *et al.*, 2007; Refulio-Rodriguez *et al.*, 2012; P. M. Peterson and R. J. Soreng, unpubl. res.) from humid to montane forests in the Andes (from Bolivia to Colombia); its unusual two- to three-flowered spikelets have minute or small glumes, mucronate lemmas with two lobes beside the mucro, and florets separated on long flexuous rachilla internodes. Sub-clade G includes a morphologically and ecologically diverse group of four species, ranging from dwarf (*P. humillima*), to slight and medium-sized (*P. marshallii*), to robust plants up to 1 m tall (*P. fibrifera* and *P. ramifera*). All of these grow in Peru (with *P. humillima* also in Argentina and Bolivia), although they are distributed at different altitudes and occupy different environments. *Poa fibrifera* is probably sequentially adjusted gynomonoecious; the other three species are simple gynomonoecious.

Sub-clade E includes a set of dwarf high Andean species that are mostly exclusively pistillate species. These exclusively pistillate species have stamens reduced to staminodes in all plants over large geographic ranges, except in *P. aequigluma* and *P. gymnantha*, where plants in a few localities have well-developed, fertile-appearing, long anthers. Sub-clade E is characterized by plants 2–8 cm tall (sometimes taller in *P. gymnantha*), with mostly (one) two florets per spikelet, inhabiting humid prairies, vegas and dry steppe at high elevations [(3000–) 4000–5000 m a.s.l.] in the Altoandean province. The simple gynomonoecious species *P. lepidula* (Nees & Meyen) Soreng & L.J. Gillespie (sect. *Anthochloa*), a dwarf of the highest sparsely vegetated elevations in the Andes (with 2–7 flabellate florets per spikelet) and the gynodioecious *P. unispiculata* are firmly nested within sub-clade E, a relationship previously detected by Refulio-Rodriguez *et al.* (2012). The gynomonoecious species, *P. linearifolia* Refulio (previously as *Dissanthelium longifolium* Tovar) from high elevations in Peru, is sister to sub-clade E. Optimization of the breeding system shows a probable simple gynomonoecious origin for sub-clade E.

### Breeding system evolution

Hermaphroditism appears as the most primitive state, and diclinism is derived (Fig. 3A, B). Dicliny is rare in sub-tribe Poinae and surrounding sub-tribes, and only *Poa* exhibits gynomonoecy within the subfamily (Connor, 1981).

The phylogeny helps to infer at least three major pathways from hermaphroditic ancestors to diclinism (Figs 3 and 4). Two major evolutionary pathways indicate a direction to dioecy, one in South America (probably via gynodioecy) and one in North America; a third pathway leads from hermaphroditism to simple gynomonoecism in Andean species of South America. Two derived states evolved independently from hermaphroditic, gynomonoecious or dioecious species. The step leading to strictly pistillate flowering probably included total supression of maleness in flowers and gain of apomixis, allowing species to perpetuate themselves in extreme habitats and produce seed without the need for pollen to stimulate endosperm development. The occurrence of sequentially adjusted gynomonoecy is possibly due to the suppression of maleness in space (partial or whole inflorescence) and in time (developmentally and through the growing season); Figs 2 and 4.

Dioecism appears to have originated twice in the Americas, independently in both hemispheres, representing dissimilar pathways (Figs 3–6). In one case, gynodioecy appeared as an intermediate state to dioecy in the South American *Dioicopoa s.l.* In the second case, dioecy appears suddenly in the North American *Madropoa* with no obvious intermediate states (Fig. 3). Soreng (1991, 2000) and Soreng and Keil (2003) suggested that dioecy in *Madropoa* evolved via intermediate pathways, proceeding from sequentially adjusted gynomonoecism [found in the *P. nervosa* complex of *Madropoa* (*sensu* Soreng, 2007)] to gynodioecy, or to sub-dioecy. A review of phylogenetic studies showed that dioecy can be gained and lost several times once evolved, and that there are more genera with hermaphroditic and dioecious species than genera with intermediate breeding systems (Renner 2014), suggesting possible evidence for this evolutionary pathway. We would emphasize that diagnosing intermediate stages is not a trivial matter, and these may have been overlooked in broad surveys, especially those dependent mainly on herbarium material. However, considering that less than half of section *Madropoa* was sampled in this study (Table 3), it will be critical to include a full set of *Madropoa* species to confirm this hypothesis.

Simple gynomonoecism could have originated twice from hermaphroditic ancestors in South American and Mesoamerican *Homalopoa*. This breeding system also represents an intermediate step towards the evolution of strictly pistillate apomictic populations in extreme habitats of the High Andes. Only the presumed gynodioecious species *P. unispiculata* (Davidse *et al.*, 2010) resolved within the pistillate sub-clade E reveals a pathway from gynomonoecy to gynodioecy. An alternative hypothesis of gynodioecy as a precursor condition leading to the development of pistillate apomictic populations (Fig 3A) needs further exploration of the reproductive biology and ecological adaptations in this clade. In *Poa*, phenotypic plasticity (Frenot *et al.*, 1999; Couso and Fernández, 2012) and polyploidy are frequent phenomena that, in conjunction with apomixis, favour the adaptability of pistillate populations, thus fixing genetic variation established via earlier sexual reproduction to maintain successful genotypes in extreme climates with short flowering seasons (Asker and Jerling, 1992). Advanced dicliny may also favour the establishment of pistillate apomictic reproduction when population sizes are low and the chance of pollen meeting stigma is critically reduced, as hypothesized for sect. *Madropoa* species (Soreng, 2000).

Direct relationships among dioecious and gynodioecious species and exclusively pistillate species are rare (except in sect. *Madropoa*). Species with only pistillate plants appear independently several times in the phylogeny. In addition to pistillate species of sub-clade E, the Peruvian–Bolivian pistillate species resolved in sect. *Dioicopoa s.s.* is the only pistillate taxon in this dioecious clade and is geographically isolated from the other taxa in the section. Within sect. *Madropoa* (clade C), *P. cusickii* has two alpine subspecies (subspp. *epilis* and *purpurascens*) that are exclusively pistillate, and *P. wheeleri* is almost always pistillate. The other two subspecies of *P. cusickii* have both sexually reproducing (gynodioecious and dioecious) and pistillate–apomictic populations, as do the three subspecies of *P. fendleriana* (dioecious), and populations of *P. pringlei* Scribn. (dioecious in the Oregon and California Coast Ranges only, and pistillate elsewhere) (Soreng and Van Devender, 1989; Soreng, 1991, 2000).

As shown in Fig. 3A and B, reversal to hermaphroditism is only represented in the *Tovarochloa* + *Aphanelytrum* clade and in *P. acinaciphylla*; the first derived from gynomonoecy while *P*. *acinaciphylla* is resolved among gynodioecious ancestors. In our phylogeny, reversal to hermaphroditism could be explained through different mechanisms: by the loss of the terminal pistillate floret in sect. *Tovarochloa*, by a reversal to the expression of maleness in the upper floret of *Aphanelytrum* spp. and by the loss of male suppression in pistillate plants of *P. acinaciphylla*.

### Dating *Poa* and the evolution of the breeding system in New World *Homalopoa*

According to our results, *Poa* is estimated to have originated around 17·6–9·9 Mya during the Middle Miocene (Fig. 6). Eurasia is considered to be the centre of diversification of the genus (Hartley, 1961; Soreng *et al.*, 1990; Anton and Connor, 1995), particularly the western part where most of the sections and diploids occur (Soreng *et al.*, 2010). Although subg. *Sylvestres*, which is endemic to North America today, is sister to the rest of the genus (excluding *Arctopoa*), Soreng (1990) suggested that *Sylvetres* might have originated from an early dispersal from the European sub-continent when closer to North America. However, there is evidence from reticulate origins of *Aniselytron* and *Arctopoa* with ancestral *Sylvestres* in East Asia (Gillespie *et al.*, 2010), which would be consistent with an early dispersal to North America via Beringia for *Sylvestres*. The origin of *Poa* supersect. *Homalopoa*, a diversified group occurring today in Africa, Australasia, Eurasia, North America and South America, is suggested to have been in Eurasia 8·4–4·2 Mya ago. The expansion of *Homalopoa* to the Americas also occurred through Beringia, with colonizing opportunities favouring expansion through North and South America (Soreng *et al.*, 2010). Because our results show a single New World *Homalopoa* clade, there was presumably a single dispersal event for this group from Eurasia to North America; this ancestor of the New World *Homalopoa* then rapidly diversified and radiated in the Late Miocene–Early Pliocene (8–4 Mya). Analyses including additional species of *Homalopoa* from Eurasia are needed to test this hypothesis.

Several major geological and environmental events facilitated dispersal to South America and promoted the rapid radiation of *Homalopoa* in both South and North America. During the Middle Miocene, a widespread area of South America previously flooded by the ‘Paranean Sea’ and the ‘Tethys Waterspout’ was succeeded by plains that extended north from northern Patagonia to central and northern Argentina, Uruguay, along the eastern slopes of the rising Andes of northern Bolivia, southern Peru and Venezuela, and also in the upper Amazon basin (Pascual *et al.*, 1996). During this period known as ‘the Age of the Southern Plains’ (Pascual and Bondesio, 1982; Ortiz-Jaureguizar and Cladera, 2006), the climate was cooler, seasonality was marked, and more varied environmental sub-division occurred (Pascual *et al.*, 1996); consequently, new opportunities were established for colonization of grasses in South America (Simpson, 1983). Although the Isthmus of Panama connected North and South America about 4 Mya ago, partly due to sea level drop from glaciation cycles, *Poa* presumably crossed between the continents on tropical mountain tops or longer distance hitchhiking via birds. The tangled hairs on the lemma nerves and long tufts of hairs on the callus of *Poa* are quite effective for seed dispersal by animals (Davidse, 1987), but transporting cool temperate species across the tropical lowlands via mammals seems improbable, leaving birds and winds as the most likely vectors for migrations between North and South America. The uplift of the Andes in South America occurred during the Middle Miocene through Early Pliocene until reaching modern elevations by around 2·7 Mya (Gregory-Wodzicki, 2000). Similarly in North America, regional uplift of major mountains, such as the Sierra Nevada in western North America, and local doming that deformed the peneplain, such as the San Juan Formation (4 Mya), occurred in the transition from Tertiary to Quaternary time (Wallace and Kirtley, 1932; Ruddiman and Kutzbach, 1991). In both hemispheres, mountain uplifts brought severe consequences to landscapes and vegetation, exposing species to drastic climatic and geological changes in a relatively short time. During the Pleistocene (about 2·5 Mya), glacial cycles similarly affected landscapes and life. New habitats, fragmentation of habitats, aridization and colder climates probably favoured speciation in the New World *Homalopoa*. All principal lineages described in this study, such as sections *Dioicopoa*, *Madropoa*, *Dissanthelium*, *Tovarochloa*, clade F and the dwarf sub-clade E, diversified in parallel at about the same time during the Pliocene to early Pleistocene (5–2 Mya) in North and South America. At the same time, speciation of major lineages also took place in Australasia, where the ancestor of sect. *Brizoides* diversified (4·5–0·99 Mya) in agreement with a suggested rapid diversification in the Pleistocene for this Australian group (Gillespie *et al.*, 2009; Hoffmann *et al.*, 2013; Griffin and Hoffmann, 2014), and coincident with the appearance of prominent grasslands or shrubland/grassland mosaic vegetation dating from the mid-Pliocene (Birch *et al.*, 2014).

The evolution of major New World lineages within *Homalopoa* may have been facilitated by the diversification of the breeding systems that helped to perpetuate successful genotypes in extreme conditions, particularly at higher altitudes of both hemispheres. Possibly dicliny evolved under pressure to circumvent inbreeding depression once the hermaphroditic and self-compatible ancestor(s) established in the New World. Gynomonoecious lineages appear to have given rise to the Altoandinean–Puneña clades (E, F); in sub-clade E, apomictic, strictly pistillate species favoured the establishment of adaptive characteristics to extreme habitats. Most probably, dioecy appeared independently in North and South America with the diversification of *Madropoa* in mountains of the western USA (5·60–2 Mya) earlier than *Diocopoa* (3·2–1·24 Mya) in Sub-antarctic and Patagonian regions in southern South America. Gynodioecious species represent intermediate pathways to dioecy (clade D+), and also originated independently as derived states several times in the evolution of New World *Homalopoa* (such as *P. lilloi* and *P. unispiculata*).

Few long-distance dispersal events are the best explanation for the position of some species in the phylogeny. *Poa reitzii* would have dispersed from North to South America, and *P. palmeri* in the opposite direction during the Early Pliocene; later, during the Pleistocene, another long-distance dispersal event took the ancestor of *P. arachnifera* from South to North America. These long-distance dispersal events in dioecious species of *Poa* were most probably related to the migration of birds, or facilitated by wind dispersal of hairy seeds.

### Conclusions

Based on a comprehensive examination of many taxa of *Poa*, Anton and Connor (1995) proposed evolutionary hypotheses for the diversification of the breeding system that our phylogeny, for the most part, supports. However, the timing and selective pressures favouring the different sexual pathways in *Poa*, from hermaphroditism to gynomonoecy and to dioecy, are suggested here for the first time based on a robust dated phylogeny. Diversification of the breeding system in the New World *Homalopoa* occurred at the end of the Miocene and Early Pliocene. As a consequence of the formation of the Panama Isthmus and mountain uplifts in tropical latitudes, connection between continents and the formation of new habitats favoured the expansion of the ancestral species of New World *Homalopoa*. During the Pliocene, diversification of breeding systems helped to perpetuate genotypes in extreme climatological and environmental conditions. Later, long-distance dispersal events are the best explanation for species to migrate between North and South America, explaining the presence of species from North and South America in the same clades. Speciation accompanied cycles of glaciations during the Pleistocene, giving rise to the extensive variation in morphology and breeding system among species of major lineages found in the New World *Homalopoa*.

Finally, because the evolutionary history of *Poa* is linked to the diversification of the breeding system in the Americas, infrageneric taxonomic categories can be accommodated to the phylogenetic results in correlation with the type of reproductive system. A new sectional treatment is needed to classify the gynomonoecious species of clade E+, the dwarf and pistillate species of the South American Punapoa group (sub-clade E) and the group of Andean species of clade F (Table 3), and to reclassify species of sects *Acutifoliae*, *Dasypoa* and *Homalopoa s.l.* In addition, some species need to be realigned in well-supported monophyletic sections, while further work is needed to place other species included in polytomies.

Our study is an important contribution to the understanding of the evolutionary patterns in *Poa* associated with the sexuality of species, current distribution and historical biogeography; the future challenge is to incorporate all this information into taxonomic categories that reflect evolution within the New World Poas.

## SUPPLEMENTARY DATA

Supplementary data are available online at www.aob.oxfordjournals.org and consist of the following. Figure S1: majority rule consensus tree of *Poa* obtained from the 100 000 most parsimonious trees based on four molecular markers: ITS, ETS, *trnT*-L and *trnL*-F. Figure S2: Bayesian 50 % majority rule consensus tree of the combined data matrix using ITS, ETS, *trn*T-L and *trn*L-F sequences from species of *Poa* and related taxa. Appendix S1: voucher information for species used in the phylogenetic and dating analyses. Appendix S2: geographic coordinates of the specimens used to represent species in their geographic range.

Supplementary Data
