# Supplementary material for: Breeding system diversification and evolution in American Poa supersect. Homalopoa (Poaceae: Poeae: Poinae)
Source: Ann Bot. 2016 Jul 3;118(2):281–303. doi: 10.1093/aob/mcw108 (PMC4970369; doi:10.1093/aob/mcw108)
Supplement: Supplementary Data [file supp_118_2_281__index.html]

Supplementary Data 

# Breeding system diversification and evolution in American *Poa* supersect. *Homalopoa* (Poaceae: Poeae: Poinae)

## Supplementary Data

files

- Supplementary Data - docx file
- Supplementary Data - xlsx file
