# Supplementary material for: Breeding system diversification and evolution in American Poa supersect. Homalopoa (Poaceae: Poeae: Poinae)
Source: Ann Bot. 2016 Jul 3;118(2):281–303. doi: 10.1093/aob/mcw108 (PMC4970369; doi:10.1093/aob/mcw108)
Supplement: Supplementary Data [file supp_mcw108_aob-15932-s01.docx]

APPENDIX 1. Voucher information for species used in the phylogenetic and dating analyses: Infrafamiliar classification, tribal and infrageneric classification only for Pooideae and *Poa* species, respectively; species name; asterisks indicate species used in the dating analysis; character states of the breeding system used in tree optimization followed by coding assigned; voucher and herbarium information for new sequences; GenBank accession numbers given in the following order: ITS, ETS, *trn*T-L (or *trn*T-L-F when provided all together); *trn*L-F. Hyphen indicates absence of data.

**Subfamily Bambusoideae**: *Chusquea latifolia* L.G. Clark, *, AF019788, -, KJ870990, HQ292315; *Phyllostachys nidularia* Munro, *, DQ131503, -, DQ137333. **Subfam. Chloridoideae**: *Astrebla lappacea* (Lindl.) Domin, *, Hilu 5639 (VPI), AY576668, -, AY576672, -; *Eragrostis curvula* (Schrad.) Nees, *, DQ655813, -, JQ972990, DQ655870; *Vaseyochloa multinervosa* (Vasey) Hitchc., *, Hilu 5699 (VPI), AY576671, -, AY576675, -. **Subfam. Danthonioideae**: *Cortaderia fulvida* (Buchanan) Zotov, *, AF367615, -, GQ471369, EU401167. **Subfam. Panicoideae**: *Panicum miliaceum* L., *, AY129713, -, JQ972982, JQ972957; *Paspalum dilatatum* Poir., *, KF163842, -, DQ104323, DQ104303; *Thysanolaena maxima* (Roxb.) Kuntze, *, AF019854, -, JQ972980, GQ869909. **Subfam. Pharoideae**: *Pharus latifolius* L., *, AF019786, -, FJ644169, EF137587. **Subfam. Pooideae**, tribe Brachypodieae: *Brachypodium distachyon* (L.) P. Beauv., *, -, -, -, JX665601, -, DQ336855, JX665890; tribe Bromeae: *Bromus tectorum* L., *, -, -, -, L36485, -, KF600709, -; tribe Hordeeae: *Hordeum vulgare* L., *, -, -, -, AF438195, -, KF600708, -; *Triticum turgidum* L., *, -, -, -, AY450266, -, JX456363, -; tribe Meliceae: *Glyceria máxima* (Hartm.) Holmb., *, -, -, -, FJ013226, -, EU119366, EF137603; tribe Poeae, subtribe: Agrostidinae: *Agrostis mertensii* Trin., *, -, -, Gillespie 6802 (CAN), GQ324467, GQ324236, DQ353956, -; subtribe Alopecurinae: *Alopecurus magellanicus* Lam., *, hermaphroditic, 1, Gillespie *et al.* 6576 (CAN), EU792345, GQ324237, DQ353966, -; subtribe: Anthoxanthinae: *Anthoxanthum montícola* subsp. *alpinum* (Sw. ex Willd.) Soreng, *, -, -, Gillespie & Consaul 6859 (CAN), EU792323, GQ324241, DQ353953, -; subtribe Aveninae: *Avena longiglumis* Durieu, *, -, -, -, DQ539597, -, DQ631529, DQ631463; *Helictotrichon sempervirens* (Vill.) Pilg., *, -, -, Soreng 4622 (US), EU792325, GQ324269, DQ353955, -; *Koeleria albescens* DC., *, -, -, -, DQ336824, -, DQ336870, DQ336844; subtribe Brizinae: *Briza media* L., *, -, -, -, DQ539583, -, DQ631512, DQ631446; subtribe Dactylidinae: *Dactylis glomerata* subsp. *hispanica* Roth, *, -, -, Soreng 3682 (US), KM523776, KM523700, DQ353961, -; subtribe Holcinae: *Deschampsia brevifolia* R. Br., *, -, -, Gillespie & Consaul 6810b (CAN), EU792328, GQ324262, DQ353962.2, -; subtribe Loliinae: *Festuca baffinensis* Polunin, *, -, -, Gillespie & Consaul 6920 (CAN), GQ324476, GQ324268, DQ353951, DQ353952; *Festuca lasto* Boiss., *, -, -, -, AF303418, -, EF585060, AY098998; *Festuca ovina* L., *, -, -, -, AY327792, -, DQ367406, AF533063; *Festuca paniculata* (L.) Schinz & Thell., *, -, -, -, AF303407, -, DQ336858, AF533050; *Lolium perenne* L., *, -, -, -, AF303401, -, EF379024, AF478504; *Vulpia unilateralis* (L.) Stace, *, -, -, -, AY118095, -, EF585130, AY118106; subtribe Miliinae: *Milium vernale* M. Bieb., *, -, -, Soreng 3748 (US), EU792340, GQ324274, DQ353963, -; subtribe Phalaridinae: *Phalaris canariensis* L., *, -, -, -, DQ539580, -, JF951100, -; subtribe Phleinae: *Phleum pratense* L. , *, hermaphroditic, 1, Soreng 4293 (BH), EU792341, GQ324284, DQ353964, -; subtribe Poinae: *Arctagrostis latifolia* (R. Br.) Griseb., *, hermaphroditic, -, Gillespie *et al.* 6586 (CAN), EU792351, GQ324245, DQ353969, -; *Arctophila fulva* (Trin.) Andersson, *, hermaphroditic, 1, Aiken 99-230 (CAN), EU792347, GQ324246, DQ354058, -; *Cinna arundinacea* L., *, -, -, Soreng & Olonova 7462 (US), EU792343, GQ324260, EU792436, -; *Hookerochloa hookeriana* (F. Muell. ex Hook.f.) E.B. Alexeev, *, -, -, Jacobs 9127 (NSW), EU792348, GQ324272, EU792435, -; *Nicoraepoa andina* (Trin.) Soreng & L.J. Gillespie, *, gynodioecious, 3, Soreng & Soreng 7182 (US), EU792354, GQ324275, DQ353971, -; *Nicoraepoa chonotica* (Phil.) Soreng & L.J. Gillespie, *, gynodioecious, 3, Peterson *et al.* 17301 (US); Soreng & Soreng 7309, GQ324276, KU763379, KU763463, -; *Poa* subg. incerti cedis, *Poa hachadoensis* Nicora var. *hachadoensis*, -, hermaphroditic, 1, Peterson *et al.* 17409 (US), KU756516, KU763412, KU763492, -; *Poa hachadoensis* var.*pilosa* Nicora, *, hermaphroditic, 1, Peterson *et al.* 19188 (US), KU756517, KU763413, KU763493, -; *Poa mendocina* Nicora & F.A.Roig, *, hermaphroditic, 1, Peterson *et al.* 19202 (US), KU756536, KU763432, KU763510, -; Sect. *Secundae*: *Poa secunda* subsp. *secunda* J. Presl, -, hermaphroditic, 1, Soreng 5812 (US), EU792393, KU763450, DQ353991, -; *Poa stenantha* Trin., -, hermaphroditic, vivs, 1, Soreng & Soreng 6068-1 (US), KU756554, KU763455, DQ354057, -; *Poa* subg. Ochlopoa: sect. *Alpinae*: *Poa alpina* L., *, hermaphroditic, vivs, 1, Alberta (SK 236892), KU756495, KU763387, KU763470, -; sect. *Parodiochloa*: *Poa flabellata* (Lam.) Raspail, -, monoecious (terminal flowers pistillate), 6, Stoney Wright (RNSG4), EU792380, GQ324320, DQ353982, -; *Poa* subg. *Poa*: supersect. *Homalopoa*: "Punapoa" group: *Poa aequigluma* Tovar., -, pistillate/(possibly gynodioecious), 2, 3, Negritto *et al.* 87 (CORD), KU756491, KU763383, KU763466, -; *P. aequigluma*, *, pistillate/(possibly gynodioecious), 2, 3, Peterson *et al.* 21684 (US), KU756492, KU763384, KU763467, -; *Poa chamaeclinos* Pilg., *, pistillate, 2, Negritto & Isella Meneses 128 (CORD), KU756502, KU763397, KU763477, -; *Poa gymnantha* Pilg., *, pistillate (hermaphroditic or staminate in *P. ovate* type), 2, Peterson & Soreng 15730 (US), KU756515, KU763411, KU763491, -; *P. gymnantha* Pilg., -, 2, Peterson & Soreng 15656 (US), EU792417, GQ324325, EU792462, -; *Poa humillima* Pilg., *, monoecious (terminal flowers pistillate), 6, Negritto *et al.* 61 (CORD), KU756524, KU763420, KU763500, -; *Poa marshallii* Tovar., *, monoecious (terminal flowers pistillate), 6, Peterson *et al.* 21546 (US), KM523799, KM523726, KM524086, -; *Poa perligulata* Pilg., *, pistillate, 2, Peterson *et al.* 20414 (US), KU756545, KU763442, KU763519, -; *Poa unispiculata* Davidse, Soreng & P.M. Peterson, *, gynodioecious, 3, Peterson *et al.* 20382 (US), KU756558, KU763459, KU763534, -; *Poa* supersect. *Homalopoa* sect. *Acutifoliae*: *Poa acinaciphylla* E. Desv., *, hermaphroditic, 1, Soreng & Soreng 7169 (US), JF904812, KU763380, DQ354023, -; *Poa acinaciphylla* E. Desv., -, hermaphroditic, 1, Mieres 3306 (CONC 165772), KU756489, KU763381, KU763464, -; *Poa planifolia* Kuntze, *, gynodioecious, 3, Soreng 7164 (CONC), KU756547, KU763445, KU763522, -; *P. planifolia* Kuntze, -, gynodioecious, 3, Peterson *et al.* 19233 (US), KM523800, KM523727, KM524087, -; sect. *Anthochloa*: *Poa lepidula* (Nees & Meyen) Soreng & L.J. Gillespie, *, monoecious (terminal flowers pistillate), 6, Peterson *et al.* 18138 (US), EU792422, GQ324344, EU792464, -; sect. *Brizoides*: *Poa cockaynaiana* Petrie, *, hermaphroditic, 1, Lloyd s.n. (OTA 058921), GQ324497, GQ324304, GQ324409, -; *Poa drummondiana* Nees, *, hermaphroditic, 1, Peterson *et al.* 14504 (US), EU792411, GQ324314, DQ354013, -; *Poa fax* Willis & Court, *, hermaphroditic, 1, Jacobs 9339 (NSW), EU792410, GQ324318, EU79246, -; *Poa labillardierei* Steud., *, hermaphroditic, 1, Gillespie *et al.* 7318 (CAN), GQ324519, GQ324341, GQ324430, -; *Poa meionectes* J. Vickery, *, hermaphroditic, 1, Gillespie & Jacobs 7316-1 (CAN), GQ324528, GQ324353, GQ324438, -; *Poa phillipsiana* Vickery, *, hermaphroditic, 1, Gillespie *et al.* 7369 (CAN), GQ324533, GQ324360, GQ324444, -; *Poa poiformis* (Labill.) Druce, *, hermaphroditic, 1, Soreng & Peterson 5911 (US), GQ324535, GQ324362, GQ324446 , -; *Poa porphyroclados* Nees, *, hermaphroditic, 1, Peterson *et al.* 14476 (US), GQ324537, GQ324364, GQ324448, -; *Poa pubinervis* (Vickery) S.W.L. Jacobs, *, hermaphroditic, 1, Peterson *et al.* 14510 (US), EU792408, GQ324371, DQ354048, DQ354049; *Poa sieberiana* Spreng, *, hermaphroditic, 1, Gillespie *et al.* 7319 (CAN), GQ324548, GQ324377, GQ324456, -; sect. *Dasypoa*: *Poa laetevirens* R.E. Fr., *, monoecious (terminal flowers pistillate), 6, Peterson & Soreng 15641 (US), KU756529, KU763425, DQ354019, -; *Poa scaberula* Hook. f., -, hermaphroditic, 1, Peterson *et al.* 15575 (US), EU792412, GQ324375, GQ324454, -; *Poa subspicata* Schrad., -, monoecious (terminal flowers pistillate), 6, Peterson *et al.* 21486 (US), KU756556, KU763457, KU763532, -; sect. *Dioicopoa*: *Poa alopecurus* (Gaudich. Ex Mirb.) Kunth, *, dioecious, 4, Peterson *et al.* 17122 (SI), KU756493, KU763385, KU763468, -; *Poa alopecurus* var. *fuegiana* (Hook. f.) D.M. Moore & Dogg., -, dioecious, vivs, 4, Soreng 7308 (SI), KU756494, KU763386, KU763469, -; *Poa arachnifera* Torr., *, dioecious, 4, Soreng & Soreng 5801 (US), GQ324486, GQ324290, DQ354021, -; *Poa bergii* Hieron., *, dioecious, 4, Iannone 12 (SI), KU756498, KU763392, KU763473, -; *Poa bonariensis* (Lam.) Kunth, *, dioecious, 4, Giussani & Anchorena 211 (SI), KU756499, KU763393, KU763474, -; *Poa calchaquiensis* Hack., *, dioecious, 4, Cialdella *et al.* 185 (SI), KU756500, KU763394, KU763475, -; *Poa cumingii* Trin., *, dioecious, 4, Soreng & Soreng 7081 (US), KU756505, KU763400, KU763480, -; *Poa denudata* Steud., *, dioecious, 4, Giussani & Morrone 349 (SI) and Soreng & Soreng 7224b (US), KU756508, KU763403, KU763483, -; *Poa dolicophylla* Hack., *, dioecious, 4, Peterson *et al.* 19441 (SI), KU756509, KU763404, KU763484, -; *Poa durifolia* Giussani, Nicora & F.A. Roig, *, dioecious, 4, Giussani & Morrone 338 (SI), KU756511, KU763406, KU763486, -; *Poa holciformis* J. Presl, *, dioecious, 4, Soreng & Soreng 7166 (US), GQ324512, GQ324330, DQ354054, DQ354055, DQ354056; *Poa hubbardiana* Parodi, *, dioecious, 4, Soreng 7130 (CORD), KU756522, KU763418, KU763498, -; *Poa huecu* Parodi, *, dioecious, 4, Peterson *et al.* 17480 (US), KU756523, KU763419, KU763499, -; *Poa iridifolia* Hauman, *, gynodioecious, dioecious, 3, 4, Morrone & Giussani 6223 (SI), KU756527, KU763423, KU763502, -; *P. iridifolia* Hauman, -, gynodioecious, dioecious, 3, 4, Giussani 351 (SI), KU756526, KU763422, KU763501, -; *Poa lanigera* Nees, *, dioecious, 4, Iannone 37 (SI), KU756530, KU763426, KU763504, -; *Poa lanuginosa* Poir., *, dioecious, 4, Giussani *et al.* 165 (SI), KU756531, KU763427, KU763505, -; *Poa ligularis* Nees ex Steud., *, dioecious, 4, Peterson *et al.* 17428 (US), KU756532, KU763428, KU763506, -; *Poa nubensis* Giussani, M.G. Fernandez & Morrone, *, dioecious, 4, Cialdella *et al.* 429 (SI), KU756538, KU763434, KU763512, -; *Poa obvallata* Steud., *, dioecious, vivs, 4, Soreng & Soreng 7229 (SI), KU756539, KU763435, KU763513, -; *Poa paposana* Phil., *, dioecious, 4, Soreng & Soreng 7071 (US), KU756542, KU763438, KU763516, -; *Poa reitzii* Swallen, -, dioecious, 4, Valls *et al.* 3132 (ICN), KU756550, KU763448, KU763526, -; *Poa schizantha* Parodi, -, dioecious, 4, Zuloaga sn (SI), -, KU763449, KU763527, -; *Poa spiciformis* var. *ibari* (Phil.) Giussani, -, dioecious, 4, Giussani & Oliva 115 (SI), KU756552, KU763453, KU763529, -; *Poa spiciformis* var. *spiciformis* (Steud.) Hauman & Parodi, *, dioecious, 4, Peterson *et al.* 17269 (US), KU756551, KU763452, KU763528, -; *Poa stuckertii* (Hack.) Parodi , *, dioecious, 4, Soreng *et al.* 7132 (US), KU756555, KU763456, KU763531, -; sect. *Dissanthelium*: *Poa calycina* ( J. Presl) Kunth (syn. *Dissanthelium calycinum* [J. Presl) Hitchc.], -, hermaphroditic (NA)/monoecious (terminal flowers pistillate) (SA), 6, Peterson & Refulio 16466 (US), EU792426, GQ324264, EU792466, -; *P. calycina*, 6, Peterson *et al.* 17923 (US), EU792425, KU763395, EU792467, -; *Poa parvifolia* Refulio (syn. *Dissanthelium brevifolium* Swallen & Tovar), -, monoecious (terminal flowers pistillate), 6, Peterson & Refulio 18043 (US), EU792424, GQ324263, EU792468, -; *Poa serpana* Refulio (syn. *Dissanthelium peruvianum* (Nees & Meyen) Pilg.), *, monoecious (terminal flowers pistillate), 6, Peterson & Soreng 15744 (US), EU792415, GQ324265, DQ354052, DQ354053; *P. serpana* Refulio, 6, Peterson & Refulio 18222 (US), EU792427, KU763451, EU792465, -; sect. *Homalopoa* *s.l*., *Poa aequatoriensis* Hac, *, monoecious (terminal flowers pistillate), 6, Laegaard 101410 (QCA), KU756490, KU763382, KU763465, -; *Poa atropidiformis* Hack., -, hermaphroditic, 1, Soreng & Soreng 7364 (US), GQ324489, GQ324293, DQ354020, -; *P. atropidiformis* Hack., -, hermaphroditic, 1, Peterson *et al.* 17138 (US), KU756496, KU763390, KU763471, -; *Poa bajaensis* Soreng , -, hermaphroditic, 1, Peterson *et al.* 15189 (US), KU756497, KU763391, KU763472, -; *Poa candamoana* Pilg., *, monoecious (terminal flowers pistillate), 6, Peterson *et al.* 21560 (US), KU756501, KU763396, KU763476, -; *Poa cucullata* Hack., *, monoecious (terminal flowers pistillate), 6, Becker & Terrones 1140 (LPB), KU756504, KU763399, KU763479, -; *Poa cuspidata* Nutt., -, monoecious (sequential gynomonoecious), 7, Soreng 4679-3 (US), KU756507, KU763402, KU763482, -; *Poa fibrifera* Pilg., *, monoecious (sequential gynomonoecious), 7, Peterson *et al.* 21907 (US), KU756512, KU763407, KU763487, -; *Poa gilgiana* Pilg., *, monoecious (terminal flowers pistillate), 6, Negritto *et al.* 94 (CORD), KU756513, KU763408, KU763488, -; *Poa glaberrima* Tovar, -, monoecious (terminal flowers pistillate), 6, Peterson *et al.* 19577 (US), KU756514, KU763409, KU763489, -; *Poa hieronymi* Hack., -, monoecious (terminal flowers pistillate), 6, Negritto *et al.* 177 (CORD), KU756518, KU763414, KU763494, -; *Poa horridula* Pilg., -, monoecious (sequential gynomonoecious), 7, Negrito & Techi 143 (CORD), KU756519, KU763415, KU763495, -; *Poa huancavelicae* Tovar, *, monoecious (terminal flowers pistillate), 6, Peterson *et al.* 21766 (US), KU756520, KU763416, KU763496, -; *Poa huancavelicae* Tovar, -, monoecious (terminal flowers pistillate), 6, Peterson *et al.* 21540 (US), KU756521, KU763417, KU763497, -; *Poa kurtzii* R.E. Fr., -, monoecious (terminal flowers pistillate), 6, Peterson & Soreng 15654 (US), EU792413, GQ324340, DQ354018, -; *Poa kurtzii* R.E. Fr., -, monoecious (terminal flowers pistillate), 6, Peterson *et al.* 19604 (US), KU756528, KU763424, KU763503, -; *Poa lilloi* Hack., -, gynodioecious, 3, Peterson *et al.* 19528 (US), KU756533, KU763429, KU763507, -; *Poa linearifolia* Refulio (syn. *Dissanthelium longifolium* Tovar), *, monoecious (terminal flowers pistillate), 6, Peterson *et al.* 21904 (US), KU756534, KU763430, KU763508, -; *Poa matris-occidentalis* P.M. Peterson & Soreng, -, hermaphroditic, monoecious (sequential gynomonoecious), 1, 6, Peterson *et al.* 19145 (US), KU756535, KU763431, KU763509, -; *Poa mulleri* Swallen (in subsect. *Papillopoa*), -, hermaphroditic, 1, Peterson 21459 (US), KU756537, KU763433, KU763511, -; *Poa occidentalis* Vasey , -, hermaphroditic, 1, Peterson *et al.* 18918 (US), KU756540, KU763436, KU763514, -; *Poa palmeri* R. J. Soreng & P.M. Peterson, *, monoecious (sequential gynomonoecious)/ s/t staminate, partially dioecious, [3, 4, 7], Peterson *et al.* 18787 (US), KU756541, KU763437, KU763515, -; *Poa pauciflora* Roem.& Schult., *, monoecious (terminal flowers pistillate), 6, Peterson *et al.* 21777 (US), KU756543, KU763439, KU763517, -; *Poa pearsonii* Reeder, -, monoecious (terminal flowers pistillate), 6, Davidson 3889 (K), KU756544, KU763441, KU763518, -; *Poa pearsonii* Reeder, -, monoecious (terminal flowers pistillate), 6, Peterson & Soreng 15676 (US), JF904854, KU763440, DQ354017, -; *Poa superata* Hack., -, monoecious (sequential gynomonoecious), 7, Peterson & Soreng 15615 (US), KU756557, KU763458, KU763533, -; sect. *Homalopoa* *s.s.*, *Poa chaixii* Vill., -, hermaphroditic, 1, Soreng 4677 (US), EU792404, GQ324299, EU854590, -; sect. *Madropoa*, *Poa chambersii* Soreng, *, dioecious, gynodioecious, 4, Soreng 5858 (US), KU756503, KU763398, KU763478, -; *Poa cusickii* var.*epilis* (Scribn.) C.L. Hitchc., *, dioecious, gynodiocious, pistillate, 2, G350-1 G80 (CAN), KU756506, KU763401, KU763481, -; *Poa douglasii* subsp. *macrantha* (Vasey) D.D. Keck , *, dioecious, 4, Soreng 2959 (US), KU756510, KU763405, KU763485, -; *Poa fendleriana* (Steud.) Vasey, *, dioecious, pistillate, 4, Gillespie 6292 (CAN), EU792403, GQ324319, DQ354027, -; *Poa macrantha* Vasey, *, dioecious, 4, Soreng 5861 (US), EU792407, GQ324348, DQ354028, -; *Poa nervosa* (Hook.) Vasey, *, monoecious (sequential gynomonoecious), 7, Soreng 5849 (US), EU792405, GQ324356, DQ354025, -; *Poa pfisteri* Soreng, -, dioecious, 4, Pfister s.n. (US), -, KU763443, KU763520, -; *Poa piperi* Hitchc., *, dioecious, 4, Soreng 5961 (US), KU756546, KU763444, KU763521, -; *Poa porsildii* Gjaerev., *, dioecious, 4, Soreng & Soreng 6147-1 (US), GQ324538, GQ324365, DQ354024, -; *Poa wheeleri* Vasey, *, pistillate (of diclinous origin), 2, Soreng 5985-2 (US), KU756559, KU763460, KU763535, -; sect. *Plicatae*, *Poa plicata* Hack., -, hermaphroditic, monoecious (terminal flowers pistillate), 1,6, Negritto & Romanutti 126 (CORD), KU756548, KU763446, KU763523, -; sect. *Tovarochloa*, *Poa apiculata* Refulio (syn. *Tovarochloa peruviana* T.D. Macfarl. & But), -, hermaphroditic, 1, Peterson & Refulio 18116 (US), EU792429, KU763388, EU792470, -; *P. apiculata* Refulio, -, hermaphroditic, 1, Refulio *et al.* 219 (RSA), EU792428, KU763389, EU792469, -; *Poa* supersect. *Homalopoa*, unplaced: *Aphanelytrum peruvianum* Sánchez Vega, P.M. Peterson, Soreng & Lægaard, -, hermaphroditic, 1, Sanchez Vega *et al.* 11718 (US), EU792421, KU763377, KU763462; *A. procumbens* (Hack.) Hack., -, hermaphroditic, 1, Peterson *et al.* 20582 (US), EU792419, KU763378, EU792459, -; *Poa ramifera* Soreng & P.M. Peterson, *, monoecious (terminal flowers pistillate), 6, Peterson & Soreng 21804 (US), KU756549, KU763447 , KU763525, -; *Poa spicigera* Tovar, *, pistillate, 2, Negritto *et al.* 76 (CORD), KU756553, KU763454, KU763530, -; sect. *Macropoa*: *Poa sibirica* Roshev. subsp. *sibirica*, -, hermaphroditic, 1, Olonova 2002-1 (CAN), EU792401, GQ324376, DQ354044, DQ354045; *Poa* subg. *Poa* supersect. *Poa* sect. *Poa*: *Poa pratensis* L. subsp. *pratensis*, -, hermaphroditic, 1, Gillespie 6291 (CAN), GQ324542, GQ324369, DQ354010, -; *P. pratensis* subsp. *alpigena* (Lindm.) Hiitonen, -, hermaphroditic, vivs, 1, Gillespie 5801 (CAN), GQ324539, GQ324366, DQ354006, -; *Poa pratensis* subsp. *irrigata* (Lindm.) H. Lindb., -, hermaphroditic, 1, Soreng & Soreng 6044 (US), GQ324541, GQ324368, GQ324559, -; *Poa yaganica* Speg., *, monoecious (sequential gynomonoecious), or gynodioecious (some monoecious plants are strictly perfect), 3, Soreng & Soreng 7351 (US), KU756560, KU763461, KU763536, -; *Poa* subg. *Stenopoa* sect. *Pandemos*: *Poa trivialis* L., -, hermaphroditic, 1, Soreng 4681-1 (US), GQ324555, GQ324387, GQ324462, -; sect. *Stenopoa*, *Poa glauca* Vahl, -, hermaphroditic, 1, Gillespie & Chatenoud 5963 (CAN), DQ354004, KU763410, KU763490, -; *Poa interior* Rydb., -, hermaphroditic, 1, Soreng 6136-1 (US), KU756525, KU763421, DQ354002, -; *Poa* subg. *Sylvestres:* *Poa marcida* Hitchc., -, hermaphroditic, 1, Soreng & Soreng 5974 (US), KM523798, KM523725, DQ353978, -; subtribe Scolochloinae: *Scolochloa festucacea* (Willd.) Link, *, -, Hjertson 381 UPS, KM523817, KM523746, KM524103, -; subtribe Sesleriinae: *Sesleria insularis* Sommier, *, -, Soreng 3889 (BH), KM523818, KM523747, DQ353957, -; *S. tenerrima* (Fritsch.) Hayek, *, -, Soreng *et al.* 7501-1 (US), EU792327, -, DQ631544, -; tribe Stipeae: *Amelichloa clandestina* (Hack.) Arriaga & Barkworth, *, -, Zuloaga 9637 (SI), KF294129, -, GU192058, GU192018; *Austrostipa nodosa* (S.T. Blake) S.W.L. Jacobs & J. Everett, *, -, Hill 5099, JF697715, -, GU192055, -; *Hesperostipa neomexicana* (Thurb.) Barkworth, *, -, Zuloaga 9662 (SI), EU489107, -, GU192060, GU192020; *Nassella arcuata* (R.E. Fr.) Torres, *, -, Cialdella 184 (SI), FJ461269, -, GU192032, DQ887440; *Pappostipa vaginata* (Phil.) Romasch, *, -, Peterson 19222 (US), EU489141, -, GU192049, EU489358; *Stipa offneri* Breistr., *, -, -, F434568, -, JQ972997, JQ972961.
